# NMR of Fully and Partially ^13^C‑Enriched Biomass Enhances Pendent Group Structural Characterization

**DOI:** 10.1021/acs.analchem.5c08043

**Published:** 2026-06-23

**Authors:** John Ralph, Guy Lippens, Marco Tonelli, Charles G. Fry, Clemens Anklin, Fachuang Lu, Vitaliy I. Timokhin, Nuoendagula, Rebecca A. Smith, Sarah Liu, Sally A. Ralph, Wu Lan, Yuki Tobimatsu, Fengxia Yue, Yanding Li, Mirko Bunzel, Mathias Sorieul, Stefan Hill, Shawn D. Mansfield, Wout Boerjan, Marc Van Montagu, Jorge Rencoret, José C. del Río, Yu Gao, Jenny C. Mortimer

**Affiliations:** † Department of Energy Great Lakes Bioenergy Research Center, Wisconsin Energy Institute, 5228 University of Wisconsin-Madison, Madison, Wisconsin 53726, United States; ‡ Department of Biochemistry, University of Wisconsin-Madison, Madison, Wisconsin 53706, United States; § New Zealand Institute for Bioeconomy Science (BSI), BSI Scion, Rotorua 3046, New Zealand; ∥ Toulouse Biotechnology Institute (TBI), Université de Toulouse, CNRS, INRAE, INSA, Toulouse 31077, France; ⊥ NMR Facility at Madison (NMRFAM), University of Wisconsin-Madison, Madison, Wisconsin 53706, United States; # Department of Chemistry, University of Wisconsin-Madison, Madison, Wisconsin 53706, United States; 7 Bruker Biospin, Billerica, Massachusetts 01821, United States; 8 US Forest Products Laboratory, Madison, Wisconsin 53706, United States; 9 Research Institute for Sustainable Humanosphere, 12918 Kyoto University, Kyoto, 611-0011, Japan; 10 State Key Laboratory of Advanced Papermaking and Paper-based Materials, 26467 South China University of Technology, Guangzhou, 510640 China; 11 BeiGene, 483658 Zhongguancun Life Science Park, Changping District, Beijing, 102206 China; 12 Karlsruhe Institute of Technology (KIT), 76131 Karlsruhe, Germany; 13 98587 University of British Columbia, Vancouver, BC Canada V6T 1Z4; 14 Department of Plant Biotechnology and Bioinformatics, Ghent University, Technologiepark 71, B-9052 Gent, Belgium; 15 Center for Plant Systems Biology, 82219 VIB, Technologiepark 71, 9052 Ghent, Belgium; 16 International Plant Biotechnology Outreach, VIB, Technologiepark 71, B-9052 Ghent, Belgium; 17 Instituto de Recursos Naturales y Agrobiología de Sevilla, 16379 CSIC, 41012 Seville, Spain; 18 Lawrence Berkeley National Laboratory, 1666 Joint Bioenergy Institute (JBEI), Emeryville, California 94608, United States; 19 School of Agriculture, Food, and Wine, Adelaide University, Adelaide, South Australia 5005 Australia

## Abstract

Traditional solution-state NMR experiments may either fail or yield unsatisfactory results when employing fully-^13^C-labeled biomass due to complications arising from ^13^C–^13^C coupling. Constant-time analogs of HSQC experiments mitigate such issues and deliver enhanced sensitivity. A rarely reported CT-HSQC-TOCSY experiment allows the proton coupling network to deliver much of the same value as the parent experiment on unlabeled or 10–15%-^13^C-labeled biomass polymers but with enhanced sensitivity. In the absence of a viable HMBC analog for long-range correlations, a relayed C–C experiment, i.e., via directly bonded ^13^C-labeled networks, enables the reliable assignment of coupled carbons, with the added advantage of correlating the more elusive quaternaries. A C–C-FLOPSY experiment takes advantage of fully-^13^C-labeled materials for mapping extensive carbon networks in the complex polymer mixtures inherent in biomass. Various pendent groups (tricin units, *cis*- and *trans-p*-coumarates, and *p*-hydroxybenzoates) that adorn lignins, and the *cis*- and *trans*-ferulates on arabinoxylan polysaccharides, are exquisitely revealed in spectra from isolated lignins or whole-cell-wall materials from maize, sorghum, and poplar.

## Graphical abstract

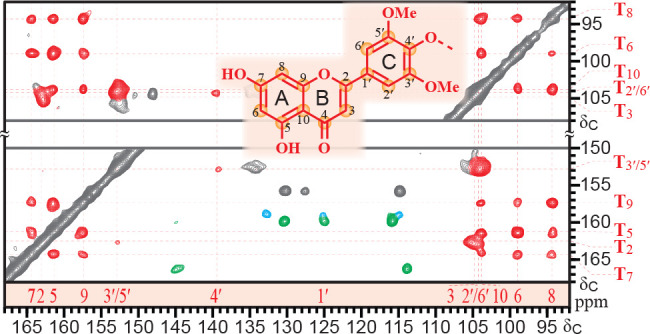

## Introduction

Nuclear magnetic resonance (NMR) spectroscopy stands as the premier method for profiling the composition and structure of the plant cell wall polymer, lignin, as reviewed.^1–4^ Informative solution-state NMR spectra can be generated from the polysaccharide and lignin components even in finely milled cell-wall (CW) materials (after solvent extraction to remove nonwall components), whether as underivatized samples simply swollen in DMSO-*d*_*6*_ or 4:1 (v/v) DMSO-*d_6_*/pyridine-*d_5_*, or after acetylation and run in CDCl_3_.^5–8^

Cultivating plants in ^13^C-enriched CO_2_ to a level of 10–15% has proven particularly advantageous for enhancing the sensitivity (signal-to-noise, S/N) of spectra and facilitating the rigorous authentication of minor components.^1,9,10^ More recently, several research groups have produced near-100%-^13^C-enriched biomass for diverse applications.^11–16^ A naïve assumption was that these materials would yield substantial S/N enhancements for NMR analysis. Such biomass was therefore highly sought-after for attempts to authenticate numerous existing plant cell wall NMR assignments and for the elucidation and documentation of previously unassigned minor structures.

Plant researchers have been disappointed to discover that the conventional experiments, so valuable for unlabeled samples with natural-^13^C-abundance levels, suffer from issues attributable to the complications arising from ^13^C–^13^C coupling. Prototypical HSQC experiments are modestly useful despite the additional coupling and the consequently unrealized sensitivity gains. Other experiments, such as HMBC, that are crucial for identifying quaternary carbons and revealing connectivity in unlabeled or moderately-^13^C-enriched lignins but relax too rapidly to be useful for CW materials, fail to provide useful correlations when using fully labeled samples. Some of the experiments developed to address full-^13^C-labeling are rather protein-specific and are not immediately applicable to deciphering the finer structure of biomass. So-called constant-time variants of the HSQC experiment^17^ mitigate some of the coupling issues, but have intensity variations resulting from differences in ^13^C–^13^C coupling constants that require multiple experiments to be run.

2D ^1^H–^13^C-HSQC-TOCSY spectra of natural polymers or those that have limited labeling are valuable for the ease with which coupling networks can be discovered. Individual structural components in a complex polymer can be rapidly identified by the usefully redundant sets of relayed correlation peaks, via the ^1^H–^1^H coupling network, when compared to the single H/C correlation peaks in an HSQC spectrum.^1,4^ We anticipated that a relayed C–C experiment, i.e., via the directly bonded ^13^C–^13^C coupling network, would similarly enable the reliable assignment of networks of coupled carbons, with the added advantage of correlating the more elusive quaternary carbons. Logically, ^13^C–^13^C correlation experiments are particularly enhanced with ^13^C-labeled samples. We report here on the value of mapping extensive carbon networks via a ^13^C–^13^C-FLOPSY (FLip-flOP SpectroscopY)^18^ variant.

As a preliminary step toward informing plant researchers about the experiments that enhance the value of NMR analysis of fully labeled plant samples, we have chosen to highlight the utility by mapping previously incompletely characterized networks for important pendent units on lignins and polysaccharides. These units (and others), as we have reviewed,^19–22^ arise from the recruitment of monomers from beyond the monolignol biosynthetic pathway, including monolignol conjugates, flavonoids, and other phenolics, into lignification. The flavone tricin, which was the first phenolic component from outside the monolignol biosynthetic pathway discovered to be involved in nucleating lignin polymerization in a subset of plants,^23–25^ serves as an illustration of the value of complete labeling and selected NMR experiments. The utility is further demonstrated via the various acids that acylate the γ–OH of lignin side-chains, including the *p*-hydroxybenzoates in dicots such as poplar (and a few monocots), and the *p*-coumarates, including the newly identified *cis-p*-coumarates, in the monocots/grasses, such as maize, sorghum, and switchgrass.^19–22,26–28^ Similarly, ferulate acylates arabinoxylan polysaccharides in monocots,^29–32^ and is clearly revealed in the spectra of CW samples. We provide examples that demonstrate the applicability of specialized NMR methods to authenticating assignments or revealing newly recognized components in isolated lignins and CW materials.

## Experimental Section

### General

Chemicals and solvents were sourced from Sigma-Aldrich (St. Louis, MO, USA) unless otherwise noted.

### Plant Materials and CW Preparation

^13^C-enriched hybrid poplar [*Populus alba × P. grandidentata*, “P39”] were grown in a self-constructed ^13^C-enrichment growth chamber as previously described.^33^ Young branch cuttings (∼5 cm) were obtained from a ∼2-year-old poplar tree and emersed in water for 2 weeks until they developed roots. The cuttings were then transferred into a hydroponic solution (Hoagland’s nutrient solution pH 6.0)^34^ and allowed to grow under a controlled ^13^C atmosphere for 68 days at which point they attained a height of ∼48 cm. Harvested poplar stems were snap-frozen in liquid nitrogen, and stored at –80 °C until further use. Stem tissues were debarked and lyophilized. ^13^C-enriched sorghum (*Sorghum bicolor*) material was obtained as previously described.^35^ Maize straw, 10% and 100% ^13^C-enriched, was obtained from IsoLife, Wageningen, The Netherlands (https://isolife.nl).

To prepare the CW material for NMR, dried plant materials were preground for 30 s in a Retsch MM400 mixer mill at 30 Hz, using stainless steel vessels (50 mL) containing a 20 mm stainless steel ball bearing. The preground material was subsequently extracted with distilled water (ultrasonication, 1 h, 3×), 80% ethanol (ultrasonication, 1 h, 3×), and finally acetone (ultrasonication, 0.5 h, 1×). Extracted biomass (up to 500 mg) was ball-milled for 4 h (interval 6 min; break 6 min; 40 cycles) using a Fritsch (Idar-Oberstein, Germany) Pulverisette 7 mill with zirconium dioxide (ZrO_2_) vessels (50 mL) containing ZrO_2_ ball bearings (10 mm × 10) spinning at 600 rpm.

### Isolation of Enzyme Lignin (EL)

The extractive-free ball-milled biomass CWs were treated with a crude cellulase (Cellulysin, EC 3.2.1.4, activity >10,000 units/g, Calbiochem) from *Trichoderma viride* to prepare the ELs. The CW materials were suspended in acetate buffer (pH 5), and 50 mg of Cellulysin was added per g of biomass. The reaction mixture was incubated on a rotary shaker at 35 °C for 48 h. The insoluble residue was collected by centrifugation (8000 rpm, 30 min), and the enzyme treatment was repeated three additional times. The pooled lignin was sonicated and washed with deionized water (3×) following enzyme treatments, and lyophilized to provide the EL. The yields of enzyme lignin from extracted ball-milled biomass, i.e., on a CW basis, were 14.2% for maize stem, 7.4% for sorghum stem, 10.2% for sorghum leaves, and 11.5% for poplar wood.

### NMR Studies

Ball-milled CW material (10–20 mg, but up to 70 mg is appropriate for lower-field or noncryoprobe-equipped instruments) or EL (10 mg) was dissolved/swollen in DMSO-*d*_*6*_ (0.6 mL) in a 5 mm external-diameter NMR tube under sonication. NMR spectra for Figures 1–3 were acquired at 300 K on a Bruker Biospin (Billerica, MA) NEO 700 MHz spectrometer equipped with a 5 mm QCI ^1^H/^31^P/^13^C/^15^N cryoprobe with inverse geometry (proton coils closest to the sample). The central DMSO solvent peak was used as the internal reference (δ_C_ 39.5, δ_H_ 2.49 ppm) in 1D spectra and the reference values (SR in Bruker TopSpin) applied to the 2D spectra. Bruker’s Topspin 5.0 software (MacOS) was used to process the acquired data. Experimental details are given below for each experiment. Full details for all of the figures are reported in the Supporting Information (SI).

**Figure 1:**
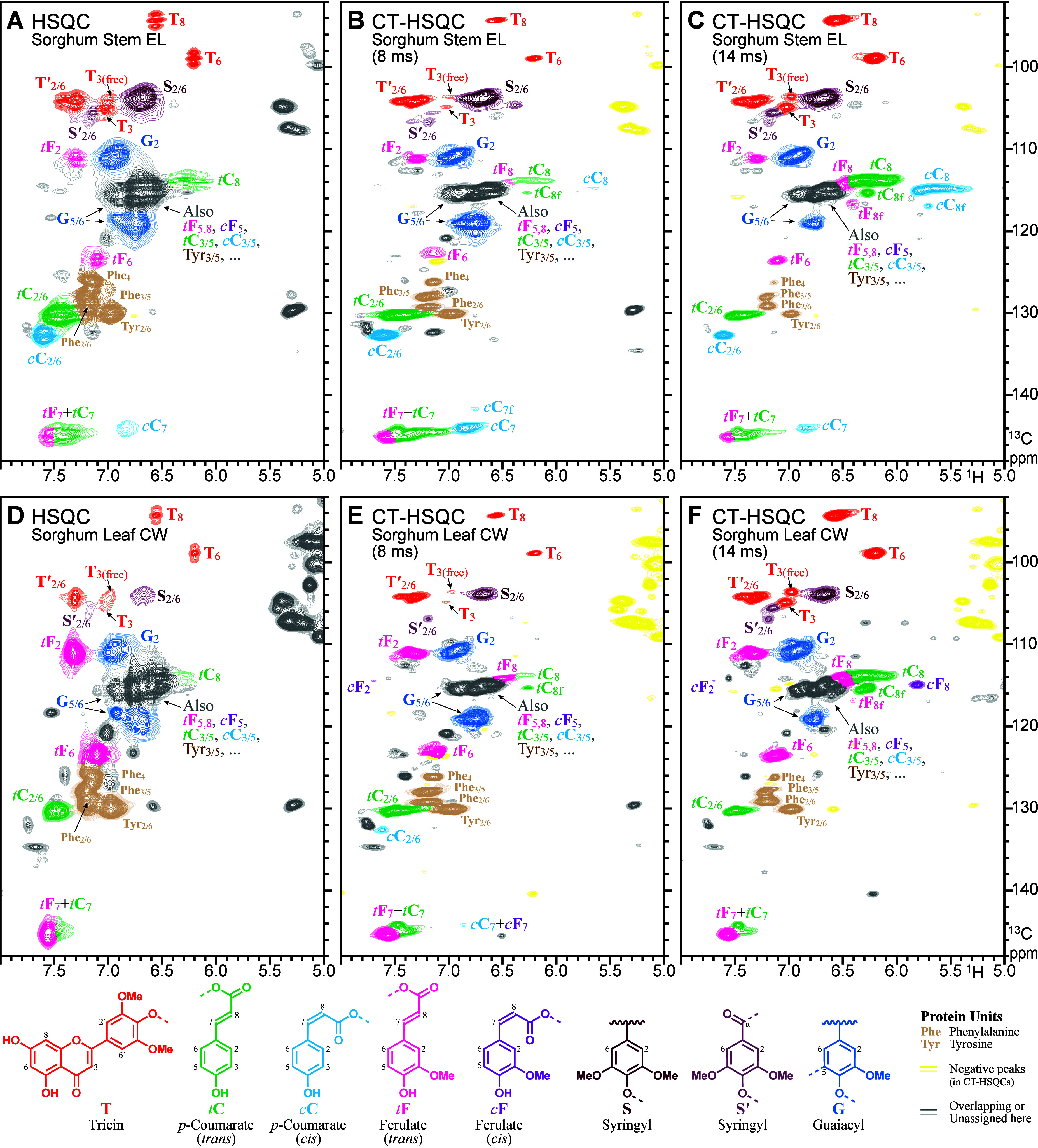
**Sorghum EL** ^**1**^ **H–** ^**13**^ **C correlation spectra.** (A) The aromatic region of a normal HSQC spectrum from fully-^13^C-labeled sorghum stem enzyme lignin (EL) illustrates the challenges arising from ^13^C–^13^C coupling.^50^ (B) The constant-time HSQC (CT-HSQC) experiment using a CT period of 8 ms. (C) Same as for B but with a CT period of 14 ms, showing the superior sensitivity for the tricin **T** peaks relative to the normal syringyl lignin **S**_2/6_ peak. (D–F) Corresponding plots from a sorghum leaf CW sample. In A–F, correlation peaks are colored to correspond to the structures below; overlapping peaks cannot be colored with complete fidelity and, as such, overlapping regions retain the gray color. The complex gray peaks centered at ∼6.7/115 ppm contain contributions from **G**_5/6_, **C**_3/5_, **F**_5_, **Tyr**_3/5_, **F**_8_, and other peaks that may be revealed in the CT-HSQC spectra (B–C, E–F). The set of lighter (40% intensity, in the same color) contours behind the darker contours are from spectra amplified 2-fold to more clearly reveal the minor peaks and, in the case of the normal HSQCs in A and D, the extent of broadening due to the ^13^C–^13^C coupling. Negative peaks in CT-HSQC data (B–C, E–F) are colored yellow.

**Figure 2:**
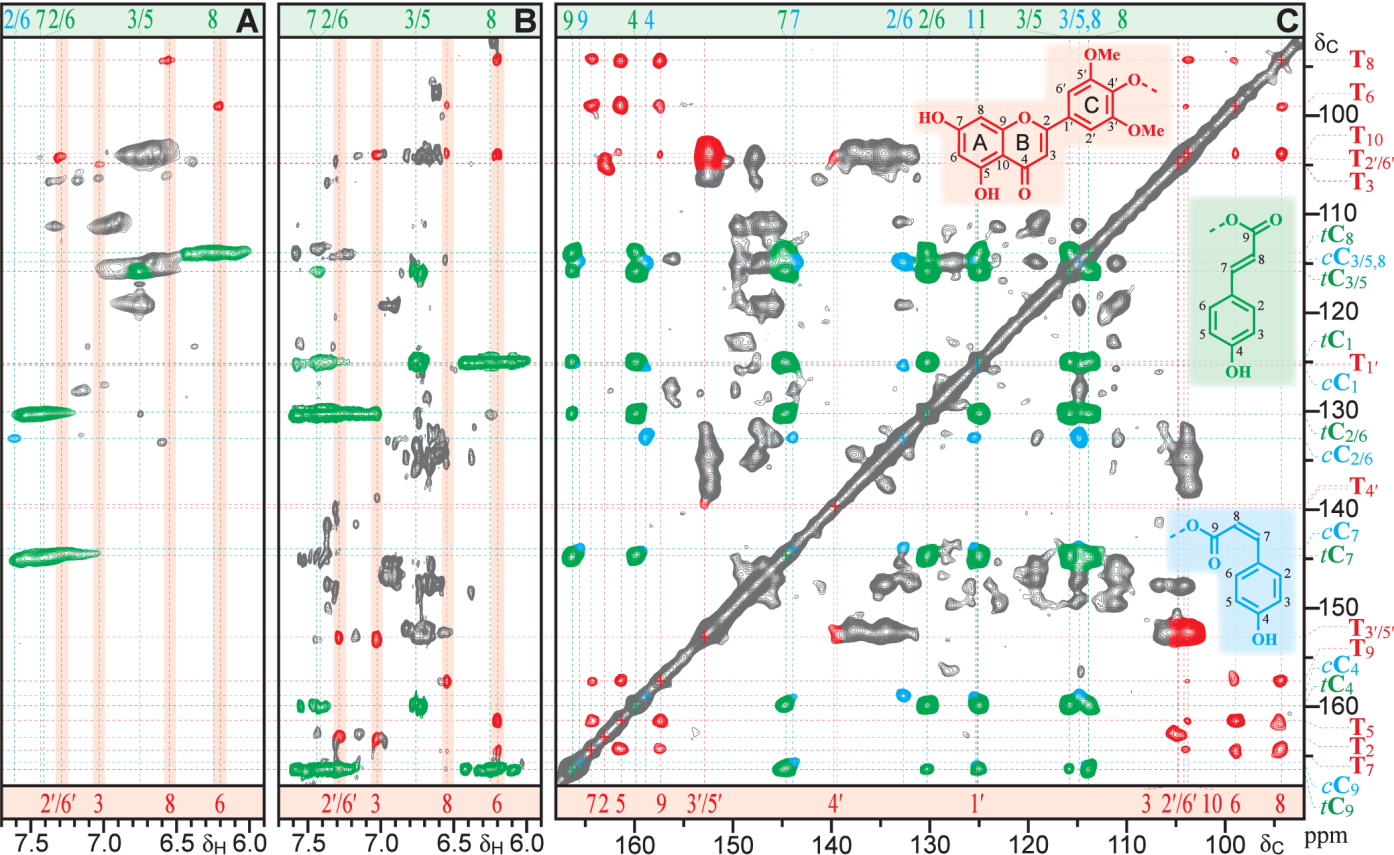
**Maize EL** ^**1**^ **H–** ^**13**^ **C and** ^**13**^ **C–** ^**13**^ **C correlation spectra.** (A) Partial HSQC spectrum from a 10%-^13^C-enriched maize stover EL that avoids the issue of ^13^C–^13^C coupling. The spectrum reveals tricin (**T**, red), *trans-p*-coumarate (*t* **C**, green), and (limited) *cis-p*-coumarate (*c* **C**, cyan) correlations. (B) Partial HMBC spectrum from the same sample as in A to help authenticate assignments. (C) Aromatic and ester carbonyl region from the C–C-FLOPSY spectrum of 100%-^13^C-enriched maize straw lignin. The spectrum shows **T** (red), *t* **C** (green), and *c* **C** (cyan) correlations. The dashed tricin assignment lines and the on-diagonal cross-marks are plotted from exact veratrylglycerol-(β–O–4′)-tricin ether model data, illustrating the excellent match with the lignin. Assignment lines for the *cis*- and *trans-p*-coumarates (*c* **C** and *t* **C**) are simply drawn through their contours.

**Figure 3:**
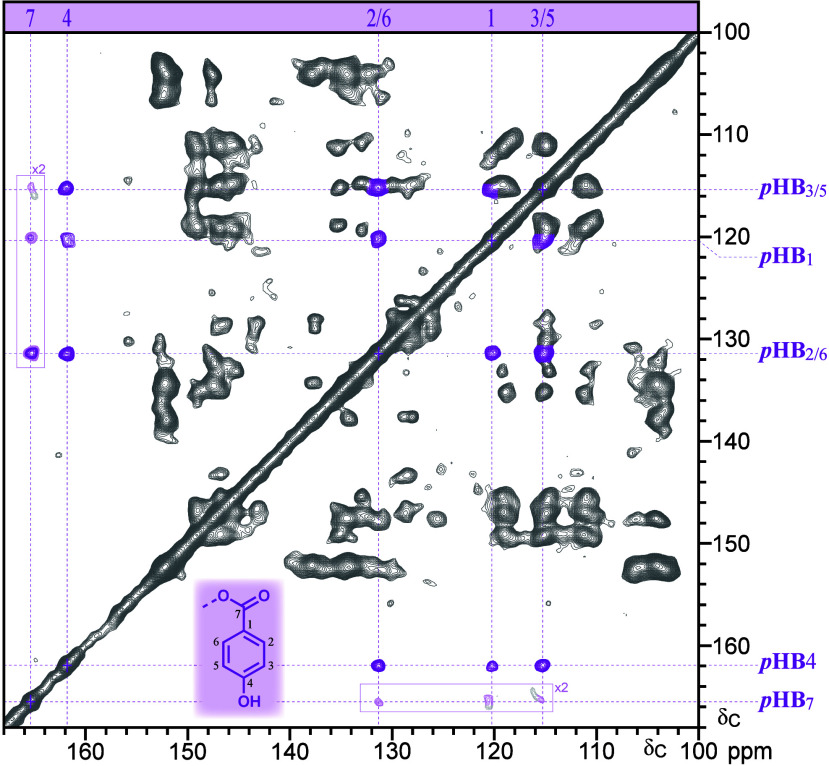
**Poplar stem EL.** Aromatic and ester carbonyl region from the C–C-FLOPSY spectrum of 100%-^13^C-enriched poplar stem lignin. The spectrum reveals the full set of *p*-hydroxybenzoate *p* **HB** (purple) correlations. Again, there is sufficient dispersion in enough of the correlation peaks that, despite congestion from lignin peaks, reliable assignment of all carbons can be made, including for the quaternary *p* **HB**_1_, *p* **HB**_4_, and *p* **HB**_7_ carbons.

#### HSQC Experiments

The normal ^1^H–^13^C correlation experiment used to acquire the spectra presented in Figures 1A, 1D, and 2A, as well as Figures S2A, S7A and S9A, was an adiabatic heteronuclear single-quantum coherence (HSQC) experiment (Bruker standard pulse sequence hsqcetgpsisp2.2, phase-sensitive gradient-edited-2D HSQC using adiabatic pulses for inversion and refocusing).^36–38^ Figure 1A was acquired from 11.66 to –0.66 ppm in F2 (^1^H) with 3448 data points (acquisition time, 200 ms) and 215 to –5 ppm in F1 (^13^C) with 1200 increments (F1 acquisition time, 15.5 ms) of 4 scans with a 1 s interscan delay; Delay d4 was 1.72 ms (1/4J, J = 145 Hz); Delay d24 was 0.89 ms (1/8J, J = 140 Hz); The total experiment time was 1.65 h. Figure 1D used the same parameters except for using 618 increments (F1 acquisition time, 8.0 ms); The total experiment time was 0.85 h. Figure 2A used the same parameters as for Figure 1D, except that 16 scans per increment were acquired, for a total experiment time of 3.37 h. Processing to 2k × 2k data points (or just 1k × 1k for the plotted spectra) typically used Gaussian apodization (LB = –0.5, GB = 0.001) in F2 and Gaussian apodization (LB = –0.2, GB = 0.001) in F1 (without linear prediction).

#### HSQC-TOCSY Experiments

The HSQC-TOCSY (Bruker standard pulse sequence hsqcdietgpsisp.2) experiments on ELs used to acquire the spectra in Figure S3D employed the same acquisition and processing parameters as for the HSQC above but with a TOCSY mixing time (d9) of 60 ms.

#### CT-HSQC Experiments

The CT-HSQC experiments used to acquire the spectra in Figures 1B–C and 1E–F, as well as Figures S2B–C, and S3B–C, were similar to Bruker’s hsqcctetgpsp.2 experiment,^17^ using pulseprogram hsqcctetgpsisp_jr.MT documented in the SI. The resolution in F1 was limited depending on the constant time (CT) period. The acquisition and processing parameters were otherwise the same as for the HSQC experiments above, with 8 scans per increment and the CT time (d23) set at 8 or 14 ms. For the CT period of 8 ms, 1052 increments (F1 acquisition time, 13.6 ms) were acquired resulting in a total experiment time of 2.89 h; for the CT period of 14 ms, 1176 increments (F1 acquisition time, 15.2 ms) were acquired resulting in a total experiment time of 3.26 h.

#### CT-HSQC-TOCSY Experiments

The CT-HSQC-TOCSY experiments used to acquire the spectra in Figures S3E–F were via the hsqcdictetgpsisp.mt2 pulseprogram documented in the SI. The parameters were the same as for the above CT-HSQC runs but with a TOCSY mixing time (d9) of 60 ms.

#### HMBC Experiments

The HMBC experiment on 10%-^13^C-labeled maize stover EL used to acquire the spectrum in Figure 2B (as also used in Figure S7B) used Bruker’s hmbcgplpndqf experiment. The spectrum was acquired from 11.644 to –0.66 ppm in F2 (^1^H) with 4096 data points (acquisition time, 237.6 ms) and 215 to –5 ppm in F1 (^13^C) with 308 increments (F1 acquisition time, 8.0 ms) of 128 scans with a 1 s interscan delay; Delay d2 was 3.45 ms (1/2J, J = 145 Hz); The long-range coupling delay d6 was 80 ms (1/2J_lr_, J = 6.25 Hz); The total experiment time was 14.56 h. Processing to 4k × 2k data points (or just 2k × 1k for the plotted spectrum) used matched Gaussian apodization (LB = –0.30, GB = 80/237.6 = 0.337) in F2 and sine-squared in F1 (using forward linear prediction with 32 coefficients).

#### C–C-FLOPSY Experiments

The C–C-FLOPSY experiment used to acquire the spectra in Figures 2C and 3, as well as in Figures S7C, S8, and S9, were via the c_ccflopsy16 pulseprogram documented in the SI. Spectra were acquired from 214.25 to –4.25 ppm in F2 (^13^C) with 4096 data points (acquisition time, 53.25 ms) and the same data-range in F1 (^13^C) with 1024 increments (F1 acquisition time, 13.3 ms) of typically 8 scans with a 3 s interscan delay; we used a spinlock time of 20 ms, based on an average J_C–C_ of 45 Hz, as discussed in the Results and Discussion section. The total experiment time was 7.11 h. Processing to 4k × 1k data points (or just 1k × 1k for the plotted spectra) used Gaussian apodization (LB = –1, GB = 0.001) in F2 and Gaussian apodization (LB = –0.5, GB = 0.001) in F1 (without linear prediction). Note that the sorghum leaf CW spectrum in Figure S8 was acquired with 96 scans per increment but with a 1s interscan delay, and had a total acquisition time of 30.36 h, but this is excessive – the usual 7.11 h experiment was already more than adequate. The total experiment times could be shortened considerably by using this shorter (1 s) interscan delay.

Test spectra were run on samples that contained 5 mM of fully labeled glucose (Figure S4) or 1,2-di-^13^C-glucose (Figure S5) in 550 μL D_2_O at 800 MHz using the same pulseprogram but with a more optimized acquisition window (62 ppm sweep width, centered at 80 ppm in each dimension). Figure S6, used to document our choice of the FLOPSY spinlock time, was recorded at 900 MHz.

### Model Compound Syntheses and NMR Data

The veratrylglycerol-(β–O–4′)-tricin ether required for plotting authenticated data-assignment lines in Figures 2, S7, and S8, was synthesized following the scheme previously outlined for the parent phenolic compound.^39^ The photochemical isomerization of 5-*O*-feruloyl-1-*O*-methyl-arabinofuranoside used for Figure S2 is described in the SI. Model compound data noted in this paper, fully assigned, are deposited in the NMR Database of Lignin and Cell Wall Model Compounds,^40^ or will be deposited in the next release in 2026.

## Results and Discussion

To obtain useful NMR spectra from fully-^13^C-labeled materials, we evaluated pulse experiments that are either not encumbered by the ^13^C–^13^C complications, or that exploit the ^13^C-enrichment and the ^13^C–^13^C coupling. Each sample is either from ball-milled CW material, or the so-called enzyme lignin (EL) isolated following the digestion of most of the polysaccharides using the mixed glycosyl hydrolases in Cellulysin;^41^ we had previously determined that this preparation contained no hydroxycinnamoyl esterase activity, allowing lignin isolation while retaining the emblematic native esters of interest in plant CW biomass.^26,42^ We avoid performing the additional dioxane-water extraction to obtain a purified lignin, so-called cellulolytic enzyme lignin (CEL),^43^ because it has lower yield and fractionates the lignin. ELs contain some polysaccharides but the entire lignin component is retained, rendering it the most representative of the *in planta* polymer.

### Short-Range (1-Bond) ^1^H–^13^C Correlation Experiments

Normal HSQC spectra serve as the gold standard for plant CW and lignin characterization. Although the HSQC_0_ experiment has been described for improved quantification in CW samples,^44–46^ the standard qualitative experiment remains simpler and continues to be extraordinarily useful for structurally characterizing polymers.^47^ For the samples used in this study, the more mobile pendent groups on the polymer are significantly over-represented, as has been noted.^8^ The aromatic region of a normal HSQC spectrum of fully ^13^C-labeled sorghum stem EL (Figure 1A) exemplifies the complexities associated with ^13^C–^13^C coupling. As illustrated by the red-colored contours in the top-middle section of the spectrum, the otherwise sharp and invariant **T**_6_ and **T**_8_ correlations from tricin units exhibit an unwelcome multiplicity – see later in Figure 2A for the appearance of such peaks in 10%-^13^C-abundance spectra, as well as in natural-abundance spectra, not shown but as initially reported.^23,24,48,49^ The triplet appearance of these peaks in Figures 1A and 1D is not directly attributable to the 1-bond ^13^C–^13^C coupling of ∼45 Hz. The wider pattern (∼130 Hz) arises from the carbon–carbon couplings that evolve during the gradients employed for the sensitivity-enhancement element.^50^ Further details on these issues are provided in the SI (Figure S1). Despite the welcome signal-to-noise improvement in easily acquired spectra, the full measure of the enhancement is not realized because all correlation peaks are similarly split and have their signals spread out over a wider range in the carbon dimension (F1). The less well-defined peaks hinder diagnostic peak assignment.

Constant-time (CT) HSQC variants were introduced over two decades ago to address the ^13^C–^13^C-coupling issue with ^13^C-labeled samples.^17^ Various sensitivity concerns and off-resonance effects were originally noted and have since been addressed.^51^ Even with the latest implementations of CT-HSQC experiments, problems arise due to the selected CT times that do not provide fully in-phase spectra, also introducing significant signal intensity distortions across the spectra.^52^ Bruker’s hsqcctetgpsp.2 pulseprogram,^17^ and the similar implementation employed here [see pulseprogram hsqcctetgpsisp.mt described in the SI for details], nevertheless demonstrate the longed-for collapse of the apparent multiplets leading to the sharpening of correlation contours in the carbon (F1) dimension and improved S/N. This is particularly exemplified in producing, in the carbon dimension, sharp **T**_6_ and **T**_8_ peaks (Figures 1B and 1C). The primary problem, not observed for the major peaks of interest in this region, is that some peaks (colored yellow) have the opposite phase. The intensities and the phase of the peaks are also dependent on the coupling constants and the chosen constant-time period (CT = n/J_C–C_, ostensibly). Acquiring two (or more) CT spectra with different CT periods is necessary to survey the peaks of interest; here we found that 8 and 14 ms spectra had their own particular advantages, even over the choice of the CT period in the “universally optimized” method described previously.^52^ A benefit of running the two CT-HSQC spectra with these CT periods is that some peaks of interest may be relatively elevated in one or the other, aiding in their assignment, tracking, and authentication.

Figure 1B depicts the sorghum stem EL spectrum with a CT period of 8 ms. Figure 1C depicts the same sample at 14 ms, demonstrating superior sensitivity for the tricin (**T**) peaks relative to the normal syringyl lignin **S**_2/6_ and guaiacyl lignin **G**_2_ peaks. A similar collapse of the multiplets in F1 is observed for the other peaks typically identified in this aromatic/double-bond region of the lignin spectra, including the usual **S** and **G** peaks, not all fully resolved, and the ferulates (**F**), and *p*-coumarates (**C**) that decorate monocot lignins. Previously unassigned peaks that are rather prevalent in this sorghum sample were only recently identified as arising from *cis-p*-coumarates (*c* **C**).^28^ The three well-resolved peaks, *c* **C**_7_, *c* **C**_2/6_, and *c* **C**_8_, are shown colored in cyan in Figure 1, with one or more of the *p*-coumarate peaks, particularly peaks *t* **C**_8_ and *c* **C**_8_, being particularly prevalent with the 14 ms CT period (Figure 1C). These *cis*-isomers likely arise from simple photochemical *trans–cis* isomerization, either during plant development or in the materials while stored in the laboratory after harvesting and isolation.^28,53^ An alternative possibility is that genes/enzymes exist *in planta* for producing the monolignol *cis-p*-coumarate conjugates employed in lignification, as for their *trans-p*-coumarate counterparts,^26^ as reviewed,^19,21,22,27^ or of a *trans–cis* isomerase as implicated in coumarin biosynthesis.^54^ Such pathway possibilities might be interesting to address. Correlation peaks for free acids are noted and annotated with an “f”, e.g., *t* **C**_8f_. It is not clear whether these are attached to the polymers, but hydroxycinnamic acid monomers are not expected to be present in the samples as the biomass was extensively solvent-extracted.

Figures 1D–1F depict analogous plots from sorghum leaf CW samples, without component isolation, to illustrate the performance of solution-state NMR experiments on ball-milled biomass that is simply swollen in DMSO-*d*_*6*_. There is minimal difference between the sorghum leaf EL sample (not shown) and the stem EL, except for a lower **S** content in the former. The primary distinction evident in the CW spectra is the significantly elevated levels of ferulate. In grasses, high levels of ferulate are present on the arabinoxylan polysaccharides,^29–32^ whereas comparatively low levels are found on lignin.^55^ Profiling the entire cell wall, with its hydroxycinnamate components on polysaccharides in addition to those on lignin, renders those ferulates particularly discernible.^7^ Spectra of these fully-^13^C-labeled sorghum leaf CW samples enable the previously unassigned *cis*-ferulate (*c* **F**) peaks to also be elucidated as described in the SI with accompanying Figure S2, thereby validating recent speculation regarding their likely presence.^28^

As for the HSQC experiment, the typically valuable 2D-HSQC-TOCSY experiment^1,4^ encounters similar challenges arising from ^13^C–^13^C coupling when applied to fully labeled samples. To address these issues, we have implemented a constant-time analog, CT-HSQC-TOCSY as detailed in the SI. On the same sorghum stem EL sample as used for Figures 1A–C, even the weak (due to the 2 Hz ^1^H–^1^H 4-bond coupling constant between the protons) correlations between the **T**_6_ and **T**_8_ peaks of tricin are effectively revealed (Figures S3D–F). This confirms their existence within the same proton coupling network.

In summary, the collapse of the multiplets incurred by the ^13^C–^13^C-coupling in the normal HSQC or HSQC-TOCSY spectra to more defined contours in their CT analogs is highly valuable. Such CT spectra on fully labeled lignins or CW samples are more closely aligned with the spectra from unlabeled or 10%-labeled materials, as demonstrated for the HSQC spectrum later in Figure 2A. The sensitivity enhancement achieved through the ^13^C-enrichment enables the ready assignment and authentication of several low-abundance components, including the *cis-p*-coumarates and *cis*-ferulates.

### Relayed ^13^C–^13^C Correlation (C–C-FLOPSY) Experiment

We developed and implemented a version of the C–C-FLOPSY experiment,^56,57^ a C–C-COSY-type experiment employing a TOCSY-alternative, a so-called FLOPSY sequence,^18^ to exploit the ^13^C-labeling for correlating carbons within the same coupling network. The C–C-FLOPSY experiment successfully demonstrates correlations between carbons in networks exhibiting normal one-bond C–C couplings that traverse all carbons within the network.

Two experimental parameters that require setting are the duration of the spin-lock time and the ^13^C B_1_ field employed for the FLOPSY pulse train. Although both parameters are constrained by the physical limitations imposed by the cryogenic probehead, their selection is primarily based on the objective of optimally connecting the carbons within the network, despite their distinct chemical shift values. Whereas previous work arrived at a 50 ms mixing time by calculating the transfer in a linear 10-spin network,^57^ we selected two samples to experimentally evaluate these parameters. The initial sample, uniformly ^13^C-labeled glucose (Figure S4), exhibited gradual connectivity between the anomeric carbon and the other carbons of the ring as the mixing time increased. At 20 ms, the complete ring in both α- and β-anomers could be mapped. The second sample consisted of glucose selectively labeled at the C1 and C2 positions. This sample simulates a pendent group in which no further transfer is feasible. We observed a maximal transfer of the C1 magnetization to the C2 position after 10 ms of mixing time (Figure S5) that subsequently returned with approximately equal values of the C1 and C2 intensities at 20 ms. As we used a 12.5 kHz ^13^C B_1_ field (corresponding to a 20 μs π/2 pulse on a 900 MHz instrument) to prepare for the requirement to cover the 138 ppm for the lignin sample, extended mixing times may lead to a warm-up of the cryoprobe. This issue was already becoming apparent at 30 ms. A comparison of C–C-FLOPSY spectra acquired at 900 MHz using 20 ms vs the ill-advised 30 ms mixing times is presented in Figure S6. The majority of the correlations are discernible in both spectra, and the few that appear to be weaker in the 20 ms spectrum, such as the correlations to *t* **C**_9_ (see below regarding Figure 2C) are clearly revealed at 20 ms on our 700 MHz instrument with the parameters specified in the Experimental Section. For systems with an average J_C–C_ of 45 Hz,^58^ we therefore adopted 20 ms as a suitable mixing time corresponding to 5 FLOPSY cycles in the c_ccflopsy16 pulse program that is documented in the SI. Utilizing the C–C-FLOPSY experiment to exploit ^13^C–^13^C correlations from a fully labeled lignin demonstrates the experiment’s value in mapping extensive carbon networks.

#### C–C-FLOPSY for Tricin Units

We were immediately captivated by the abundance of correlation peaks relating to tricin **T** using a fully-^13^C-enriched maize EL sample (Figure 2C). This strikingly useful spectrum features an array of up to five correlations to the readily identifiable **T**_6_ and **T**_8_ peaks. All of tricin’s protonated and quaternary carbons show informative and logical correlations. Quaternary carbon **T**_7_ correlates with **T**_8_, **T**_6_, **T**_10_, **T**_9_, and **T**_5_. We successfully identified all the correlations from the A and B rings of the tricin units **T**, confirming assignments of the quaternary carbons previously revealed in HMBC spectra.^23^ The dashed tricin assignment lines in Figure 2 may appear as if they were simply drawn through the various contours, but these are actually plotted from exact model data, using veratrylglycerol-(β–O–4′)-tricin ether [compounds 360 (*erythro*) and 361 (*threo*) in our NMR database].^40^ These phenol-methylated models were synthesized following the scheme previously outlined for the parent phenolic compound.^39^ Tricin units **T** are quite structurally invariant, occurring solely as 4′–O−β-linked units, but to both G and S moieties with *erythro*- or *threo*-stereochemistry.

Conventional HSQC and HMBC spectra acquired from a 10%-^13^C-labeled maize stem lignin (Figures 2A and 2B) are valuable here to illustrate the veracity of the tricin (as well as the *trans-p*-coumarate, as discussed below) assignments made in the C–C-FLOPSY spectrum (Figure 2C). It needs to be emphasized again that normal HMBC spectra fail on fully ^13^C-labeled materials, necessitating experiments on different samples with, e.g., 10% or no ^13^C-enrichment.

Figure S7C presents a spectrum resulting from sorghum stem EL, analogous to that from maize EL (Figure 2C), on the same scale. Figure S8A presents, again on the same scale, the comparative sorghum leaf EL spectrum in which the tricin **T** levels are higher, but the *p*-coumarate **C** levels (see below) are lower on a lignin basis. Figure S8B depicts the spectrum from sorghum leaf CW material, replete with its polysaccharides, enabling a comparison with that from the EL in Figure S8A. This comparison underscores the value of the C–C-FLOPSY experiment even when applied to unfractionated ball-milled biomass that has been simply swollen in DMSO-*d*_*6*_. Despite variations in cross-peak intensities and the greater congestion in the CW spectrum (Figure S8B), the range of resolved correlations remains comparable to those obtained from the more laboriously isolated EL. HMBC experiments typically fail to provide useful correlations on such CW samples due to their rapid relaxation; the NMR FID has usually completely decayed within 30 ms leaving negligible intensity after the long-range-coupling delay of 65–80 ms.

#### C–C-FLOPSY for *p*-Coumarate Units

The significance of the C–C-FLOPSY experiment is further exemplified by analyzing the pendent *p*-coumarate **C** groups on grass lignins (Figures 2C, S7C, and S8) in comparison to the conventional HSQC and HMBC spectra acquired from a 10%-^13^C-labeled maize stem lignin (Figures 2A and 2B). The *p*-coumarate contours are broad in the proton dimension due to the structural diversity of components to which they are attached. All are free-phenolic entities acylating the γ–OH of lignin side-chains, but may be associated with **G** or **S** units, and may be on *threo*- and *erythro*-isomers of β-ether units, as well as on phenylcoumarans (β–5 units), cinnamyl alcohol end groups, and other more minor structures. The comprehensive network of correlations for the **C** moieties, including many for the recently identified *cis-p*-coumarate (*c* **C**) units denoted above (Figure 1), is strikingly well revealed in the C–C-FLOPSY spectrum (Figure 2C). Indeed, had these fully-^13^C-labeled plant materials and the C–C-FLOPSY experiment been accessible to researchers, this component could have been identified and validated much earlier.

#### C–C-FLOPSY for *p*-Hydroxybenzoate Units

To further illustrate the value of the C–C-FLOPSY experiment, we also mapped the entire carbon network for *p*-hydroxybenzoate (*p* **HB**) units on fully labeled poplar lignin (Figure 3). Poplar, an angiosperm, is typically characterized by **S** and **G** units (derived from sinapyl and coniferyl alcohol). However, *Populus*, *Salix*, and *Palmae* species, as well as some seagrasses, possess *p* **HB** units that acylate the γ–OH of lignin side-chains.^19,48,59–62^ In the same manner as for tricin **T** and *p*-coumarate **C** units noted above, this relayed C–C experiment enables the reliable assignment of quaternary carbon peaks *p* **HB**_7_ and *p* **HB**_1_ that fall in a congested region of the carbon spectrum. Correlations among *p* **HB**_1_, *p* **HB**_2/6_, *p* **HB**_3/5_, *p* **HB**_4_, and *p* **HB**_7_, most of which are fully resolved from other lignin peaks, are particularly diagnostic. This spectrum, along with HSQC and HMBC spectra from an unlabeled poplar lignin, are further illustrated and discussed in the SI (Figure S9).

#### C–C-FLOPSY Authenticates the Free-Phenolic Nature of *p*-Coumarate Units and *p*-Hydroxybenzoate Units on Lignins

As previously demonstrated, phenolics devoid of additional methoxy groups on the aromatic ring, such as *p*-coumarate and *p*-hydroxybenzoate units, favor radical transfer over radical coupling reactions during lignification and therefore remain free-phenolic, i.e., pendent.^19,63^

The **C**_4_ carbons, readily and unequivocally identified in the C–C-FLOPSY spectra of grasses (Figures 2C, S7C, S8), exhibit carbon chemical shifts that align with those of free-phenolic entities and not with etherified analogs that have >1 ppm higher ^13^C chemical shifts. For instance, with the simple model compounds found in the NMR Database of Lignin and Cell Wall Model Compounds,^40^ the free-phenolic methyl *p*-coumarate (#61) has **C**_4_ resonating at 159.9 ppm in DMSO-*d_6_*, whereas its phenol-etherified analog, 4-*O*-methyl methyl *p*-coumarate (#59), has its **C**_4_ at 161.2 ppm, 1.3 ppm higher. The correlations for *t* **C**_4_ in the C–C-FLOPSY spectrum from maize (Figure 2C) or sorghum (Figure S7C) are at ∼159.8 ppm, consistent with their free-phenolic nature. More relevant etherified models have been described having data that are essentially identical to those from the simple phenol-methylated model.^64^ From the photochemically derived *cis*-isomer, the *c* **C**_4_ is again readily identified from these spectra, resonating at 158.8 ppm, in agreement with the determination obtained via HMBC experiments and exactly the same as the free-phenolic model compound, ethyl-*cis-p*-coumarate, #355.^28^ The synthesized phenol-etherified model methyl *cis*-4-*O*-methyl *p*-coumarate (the dimethyl ether of *cis-p*-coumaric acid, #353)^28^ has its C4 at 160.2 ppm, again 1.4 ppm higher than its free-phenolic counterpart.

Similarly to the *p*-coumarate units on grass lignins, *p*-hydroxybenzoates on poplars (and beyond) are also present as free-phenolic pendent units.^60^ Their free-phenolic nature is conveniently established by examining the *p* **HB**_4_ carbons that are readily identified in the C–C-FLOPSY experiment (Figure 3). Simple model compound methyl *p*-hydroxybenzoate (#18 in our database^40^) has *p* **HB**_4_ resonating at 161.97 ppm in DMSO-*d_6_*, whereas the etherified model, 4-*O*-methyl methyl *p*-hydroxybenzoate (#25), has its *p* **HB**_4_ at 163.12 ppm, 1.15 ppm higher. The correlation for *p* **HB**_4_ in the C–C-FLOPSY spectrum from poplar (Figure 3) is at ∼161.9 ppm, consistent with its free-phenolic nature.

The free-phenolic nature of *p*-coumarate and *p*-hydroxybenzoate groups have been traditionally revealed following acetylation (not shown here) after which some of the ring-carbons are predictably shifted.^40^ Without requiring derivatization, the information may be available from **C**_4_ and *p* **HB**_4_ carbons deduced from HMBC spectra, as seen for the proton-2/6 and proton-3/5 correlations to carbon **C**_4_ in the 10%-^13^C-labeled maize sample (Figure 2B), for example. However, carbons **C**_4_ and *p* **HB**_4_ are particularly effectively revealed in C–C-FLOPSY spectra (Figures 2–3, S7–S8) from these fully labeled samples, with the added advantage that these experiments perform extremely well on CW materials for which HMBC experiments often fail.

## Conclusions

Although the constant-time versions of the HSQC and possibly the HSQC-TOCSY experiments are well-known in protein research and among NMR experts, demonstrating their utility and value in obtaining more informative spectra, comparable to those from their parent analogs but with enhanced sensitivity, on fully-^13^C-labeled biomass preparations is urgently required in the biomass polymers field. The C–C-FLOPSY experiment described herein provides more readily available information from lignin polymers due to the greater range of ^13^C chemical shifts. It has significant additional value for studies on fully-^13^C-labeled biomass due to its superior applicability to readily prepared CW samples. Its shorter pulseprogram profile, resulting from the larger ^13^C–^13^C vs ^1^H–^1^H or long-range ^1^H–^13^C coupling constants, enables the acquisition of impressive spectra from rapidly relaxing samples for which HMBC experiments, in particular, fail.

## Supplementary Material



## Data Availability

The data underlying this study are available in the published article and its SI. Full Bruker TopSpin NMR data sets are available upon request

## Notes

Supplemental descriptions of NMR experiments and their pulseprograms, and 9 supplemental and complementary figures (PDF)

## ∇

School of Agriculture, Food, and Wine, Adelaide University, South Australia, 5005 Australia

## Abbreviations

NMR: Nuclear Magnetic Resonance (spectroscopy)
QCI: a proton-optimized quadruple resonance NMR inverse probe
HSQC: Heteronuclear Single-Quantum Coherence
HMBC: Heteronuclear Multiple-Bond Correlation
COSY: Correlation SpectroscopY
TOCSY: TOtal Correlation SpectroscopY
FLOPSY: FLip-flOP SpectroscopY
CT: constant-time
S/N: signal-to-noise
LB: Line Broadening (factor, Hz)
GB Gaussian Broadening (parameter: fraction of AQ, the single-scan AcQuisition time)
CW: extract-free cell walls
EL: enzymatically isolated lignins
CW: (whole) cell-wall biomass (following simple solvent-extraction to remove extractives)
DMSO: dimethyl sulfoxide
**T**: tricin
**C**: *p*-coumarate
*t* **C**: *trans-p*-coumarate
*c* **C**: *cis-p*-coumarate
**F**: ferulate
*t* **F**: *trans*-ferulate
*c* **F**: *cis*-ferulate
**FF**: photochemically derived [2+2] cyclodimer of ferulate
**FA-Ara**: 5-*O*-feruloyl-1-*O*-methyl-arabinofuranoside
*t* **FA-Ara**: *trans*-isomer of FA-Ara
*c* **FA-Ara**: *cis*-isomer of FA-Ara
**diFA-Ara**: photochemically derived [2+2] cyclodimer of FA-Ara
*p* **HB**: *p*-hydroxybenzoate
**G**: guaiacyl
**S**: syringyl. Other abbreviations are sufficiently standard.
