# Supplementary material for: NMR of Fully and Partially 13C‑Enriched Biomass Enhances Pendent Group Structural Characterization
Source: Anal Chem. 2026 Jun 23;98(26):19482–92. doi: 10.1021/acs.analchem.5c08043 (PMC13347702; doi:10.1021/acs.analchem.5c08043)
Supplement: Supplementary file 1 [file ac5c08043_si_001.pdf]

SUPPORTING INFORMATION

NMR of fully and partially ^{13}C-enriched biomass. Enhanced characterization of pendent groups

John Ralph,[1,2,3,*] Guy Lippens,[4,*] Marco Tonelli,[5] Charles G. Fry,[6] Clemens Anklin,[7] Fachuang Lu,[1] Vitaliy I. Timokhin,[1] Nuoendagula,[1] Rebecca A. Smith,[1] Sarah Liu,[1] Sally A. Ralph,[8] Wu Lan,[1] Yuki Tobimatsu,[9] Fengxia Yue,[10] Yanding Li,[11] Mirko Bunzel,[12] Mathias Sorieul,[3] Stefan Hill,[3] Shawn D. Mansfield,[1,13] Wout Boerjan,[14,15] Marc C. E. Van Montagu,[15,16] Jorge Rencoret,[17] José C. del Río,[17] Yu Gao,[18] Jenny C. Mortimer.[18,19]

[1]Department of Energy Great Lakes Bioenergy Research Center, Wisconsin Energy Institute, University of Wisconsin-Madison, Madison, WI 53726, USA.

[2]Department of Biochemistry, University of Wisconsin-Madison, Madison, WI 53706, USA.

[3]New Zealand Institute for Bioeconomy Science (BSI), BSI Scion, Rotorua 3046, New Zealand.

[4]Toulouse Biotechnology Institute (TBI), Université de Toulouse, CNRS, INRAE, INSA, Toulouse 31077, France.

[5]NMR Facility at Madison (NMRFAM), University of Wisconsin-Madison, Madison, WI 53706, USA.

[6]Department of Chemistry, University of Wisconsin-Madison, Madison, WI 53706, USA.

[7]Bruker Biospin, Billerica, MA 01821, USA.

[8]US Forest Products Laboratory, Madison, WI 53706, USA.

[9]Research Institute for Sustainable Humanosphere, Kyoto University, Kyoto, 611-0011, Japan.

[10]State Key Laboratory of Advanced Papermaking and Paper-based Materials, South China University of Technology, Guangzhou, 510640 China.

[11]BeiGene, Zhongguancun Life Science Park, Changping District, Beijing, 102206 China.

[12]Karlsruhe Institute of Technology (KIT), 76131 Karlsruhe, Germany.

[13]University of British Columbia, Vancouver, BC Canada V6T 1Z4.

[14]Center for Plant Systems Biology, VIB, Technologiepark 71, 9052 Ghent, Belgium.

[15]Department of Plant Biotechnology and Bioinformatics, Ghent University, Technologiepark 71, B-9052 Gent, Belgium.

[16]International Plant Biotechnology Outreach, VIB, Technologiepark 71, B-9052 Ghent, Belgium.

[17]Instituto de Recursos Naturales y Agrobiología de Sevilla, CSIC, 41012-Seville, Spain.

[18]Lawrence Berkeley National Laboratory, Joint Bioenergy Institute (JBEI), Emeryville, CA 94608, USA.

[19]School of Agriculture, Food, and Wine, University of Adelaide, South Australia, 5005 Australia.

Table of Contents

Introduction to the Supporting Information S2

EXTENDED FIGURE CAPTIONS – for Figures 1-3 in the main paper, with experimental details S2
Figure 1. Sorghum EL ^{1}H–^{13}C correlation spectra S2
Figure 2. Maize EL ^{1}H–^{13}C and ^{13}C–^{13}C correlation spectra S3
Figure 3. Poplar lignin EL S3

SUPPORTING EXPERIMENTS, DETAILS, AND FIGURES S4
Figure S1. Tricin T8 HSQC peaks S4
Supporting Information: Profiling ferulates via a fully-^{13}C-labeled sorghum leaf CW sample S4
Figure S2. Profiling ferulate units in sorghum leaf CW samples S5
Figure S3. Sorghum Stem EL ^{1}H–^{13}C correlation spectra S6
Supporting Information: HSQC-TOCSY experiments; CT-HSQC-TOCSY S7
Supporting Information: Short explanation of terms: spinlock time, TOCSY or FLOPSY mixing time S7
Supporting Information References (additional to those in the main text) S7
Figure S4. C–C-FLOPSY experiments on fully-^{13}C-labeled glucose S8
Figure S5. C–C-FLOPSY experiments on 1,2-di-^{13}C-labeled glucose S8
Figure S6. C–C-FLOPSY experiments at 900 MHz on the fully-^{13}C-labeled maize EL S9

Figure S7. Maize EL ^{1}H–^{13}C correlation spectra and sorghum stem EL C–C-FLOPSY spectrum S10
Figure S8. Sorghum leaf EL vs leaf CW C–C-FLOPSY spectra S11
Figure S9. Poplar lignin spectra S12

PULSEPROGRAMS S13
A) CT-HSQC pulseprogram (Marco Tonelli's version, minor editing by JR; based on Bruker's hsqcctetgpsisp) S13
B) CT-HSQC-TOCSY pulseprogram (Marco Tonelli's version) S17
C) C–C-FLOPSY pulseprogram (Guy Lippens' version, based on Eletsky et al., 2023[56]) S22

Introduction to the Supporting Information

Here we provide extended captions along with more complete experimental details for the figures in the main paper, along with additional figures and commentary to support the manuscript. Pulseprograms are included in this SI. Full Bruker TopSpin NMR datasets are available upon request.

EXTENDED FIGURE CAPTIONS – for Figures 1-3 in the main paper, with experimental details

Figure 1. Sorghum EL ^{1}H–^{13}C correlation spectra. A) The aromatic region of a normal HSQC spectrum from fully-^{13}C-labeled sorghum stem lignin (EL) illustrates the challenges arising from ^{13}C–^{13}C coupling,[50] as illustrated particularly by tricin's $\mathbf{T}_6$ and $\mathbf{T}_8$ correlations in the upper-center of the spectrum. The apparent triplet for these peaks is greater than expected from just the ~45 Hz ^{13}C–^{13}C couplings due to their evolution during the gradients used for coherence selection.[50] The spectrum was acquired with 1200 increments (F1 acquisition time, 5.5 ms) of 4 scans, with a 1 s interscan delay, for a total experiment time of 1.65 h. B) A constant-time HSQC (CT-HSQC) pulse sequence mitigates the issues with ^{13}C–^{13}C coupling producing, in the carbon dimension, sharp $\mathbf{T}_6$ and $\mathbf{T}_8$ peaks, for example. One drawback is that some peaks, identified by their yellow color coding, have the opposite phase. Another issue is that the intensities depend on the constant-time period (CT = n/$J_{C–C}$). The constant-time HSQC (CT-HSQC) experiment used a CT period of 8 ms, with 1052 increments (F1 acquisition time, 13.6 ms) of 8 scans, with a 1 s interscan delay, for a total experiment time of 2.89 h. C) As for B but with a CT period of 14 ms, showing the superior sensitivity for the tricin **T** peaks relative to the normal syringyl lignin $\mathbf{S}_{2/6}$ peak. The spectrum was acquired with 1176 increments (F1 acquisition time, 15.2 ms) of 8 scans, with a 1 s interscan delay, for a total experiment time of 3.26 h. Sorghum leaf EL spectra (not shown) were almost identical except for the lower **S** content. D-F) Corresponding plots from a sorghum leaf CW sample, *i.e.*, without component isolation, to illustrate how well the same experiments perform with unfractionated ball-milled biomass that has been simply swollen in DMSO-d_6. The main differences are the lower **S**-levels in leaves, and the greatly elevated levels of terminal ferulate **F** due to the ferulates on arabinoxylan polysaccharides in grasses, *i.e.*, when the whole cell wall replete with its polysaccharides is profiled, hydroxycinnamate components on polysaccharides, in addition to those on lignin, are more prominent than in spectra from isolated lignins. D) The spectrum was acquired with just 618 increments (F1 acquisition time, 8.0 ms) of 4 scans, with a 1 s interscan delay, for a total experiment time of 0.85 h. E) The spectrum was acquired with 8 scans per increment for a total experiment time of 2.89 h. F) The spectrum was acquired with 8 scans per increment for a total experiment time of 3.26 h. A-F) Correlation peaks are colored to correspond to the structures below; overlapping peaks cannot be colored with complete fidelity and, as such, overlapping regions retain the grey color. The complex grey peaks centered at ~6.7/115 ppm contain contributions from $\mathbf{G}_{5/6}$, $\mathbf{C}_{3/5}$, $\mathbf{F}_5$, $\mathbf{Tyr}_{3/5}$, $\mathbf{F}_8$, and other peaks that may be revealed in the CT-HSQC spectra (B-C, E-F). Correlation peaks for free acids (of unknown origin) are noted and annotated with an 'f', *e.g.*, $t\mathbf{C}_{8f}$. It is not clear whether these are attached to the polymers, but hydroxycinnamic acid monomers are not expected as the biomass was extensively solvent-extracted. The set of lighter (40% intensity, in the same color) contours behind the darker contours are from spectra amplified 2-fold to more clearly reveal the minor peaks and, in the case of the normal HSQCs in A and D, the extent of broadening due to the ^{13}C–^{13}C coupling. Negative peaks in CT-HSQC data (B-C, E-F) are colored yellow.

Figure 1 Experimental Details

Figure 1A. The ^{1}H–^{13}C correlation experiment was an adiabatic heteronuclear single-quantum coherence (HSQC) experiment (Bruker standard pulse sequence hsqcetgpsisp2.2; phase-sensitive gradient-edited-2D HSQC using adiabatic pulses for inversion and refocusing).[36-38] Acquired from 11.66 to −0.66 ppm in F2 (^{1}H) with 3448 datapoints (acquisition time, 200 ms) and 215 to −5 ppm in F1 (^{13}C) with 1200 increments (F1 acquisition time, 15.5 ms) of 4 scans with a 1 s interscan delay; Delay d4, 1.72 ms (¼J, J = 145 Hz); Delay d24, 0.89 ms (⅛J, J = 140 Hz). The total experiment time was 1.65 h. Processing to 2k × 2k datapoints (or just 1k × 1k for the plotted spectra) used Gaussian apodization (LB = −0.5, GB = 0.001) in F2 and Gaussian apodization (LB = –0.2, GB = 0.001) in F1 (without linear prediction).

Figure 1B. The CT-HSQC experiments were similar to Bruker's hsqcctetgpsisp experiment, but slightly modified by M.T. – see hsqcctetgpsisp.MT in the pulseprograms at the end of this SI. The resolution in F1 was limited depending on the constant time (CT) period. Most acquisition and processing parameters were the same as for the HSQC experiment above for Figure 1A. Acquired from 11.66 to −0.66 ppm in F2 (^{1}H) with 3448 datapoints (acquisition time, 200 ms) and 215 to −5 ppm in F1 (^{13}C) with 1052 increments (F1 acquisition time, 13.6 ms) of 8 scans with a 1 s interscan delay; Delay d4, 1.72 ms (¼J, J = 145 Hz); Delay d24, 0.86 ms (⅛J, J = 145 Hz); CT period d23, 8 ms. The total experiment time was 2.89 h. Processing to 2k × 2k datapoints (or just 1k × 1k for the plotted spectra) used fairly harsh Gaussian apodization (LB = −0.5, GB = 0.001) in F2 and Gaussian apodization (LB = –0.25, GB = 0.001) in F1 (without linear prediction).

Figure 1C. Based on parameters for Figure 1B: Acquired from 11.66 to −0.66 ppm in F2 (^{1}H) with 3448 datapoints (acquisition time, 200 ms) and 215 to −5 ppm in F1 (^{13}C) with 1176 increments (F1 acquisition time, 15.2 ms) of 8 scans with a 1 s interscan delay; Delay d4, 1.72 ms (¼J, J = 145 Hz); Delay d24, 0.86 ms (⅛J, J = 145 Hz); CT period d23, 14 ms. The total experiment time

was 3.26 h. Processing to 2k × 2k datapoints (or just 1k × 1k for the plotted spectra) used fairly harsh Gaussian apodization (LB = −0.5, GB = 0.001) in F2 and Gaussian apodization (LB = –0.25, GB = 0.001) in F1 (without linear prediction).

Figure 1D. HSQC experiments on CW materials were carried out similarly but with lower-resolution in F1 for this normal HSQC. Acquired from 11.66 to −0.66 ppm in F2 (^{1}H) with 3448 datapoints (acquisition time, 200 ms) and 215 to −5 ppm in F1 (^{13}C) with just 618 increments (F1 acquisition time, 8.0 ms) of 4 scans with a 1 s interscan delay; Delay d4, 1.72 ms (¼J, J = 145 Hz); Delay d24, 0.89 ms (⅛J, J = 140 Hz). The total experiment time was 0.85 h. Processing to 2k × 2k datapoints (or just 1k × 1k for the plotted spectra) used fairly harsh Gaussian apodization (LB = −0.5, GB = 0.001) in F2 and Gaussian apodization (LB = –0.20, GB = 0.001) in F1 (without linear prediction).

Figure 1E. Acquired from 11.66 to −0.66 ppm in F2 (^{1}H) with 3448 datapoints (acquisition time, 200 ms) and 215 to −5 ppm in F1 (^{13}C) with 1052 increments (F1 acquisition time, 13.6 ms) of 8 scans with a 1 s interscan delay; Delay d4, 1.72 ms (¼J, J = 145 Hz); Delay d24, 0.86 ms (⅛J, J = 145 Hz); CT period d23, 8 ms. The total experiment time was 2.89 h. Processing to 2k × 2k datapoints (or just 1k × 1k for the plotted spectra) used fairly harsh Gaussian apodization (LB = −0.5, GB = 0.001) in F2 and Gaussian apodization (LB = –0.25, GB = 0.001) in F1 (without linear prediction).

Figure 1F. Acquired from 11.66 to −0.66 ppm in F2 (^{1}H) with 3448 datapoints (acquisition time, 200 ms) and 215 to −5 ppm in F1 (^{13}C) with 1176 increments (F1 acquisition time, 15.2 ms) of 8 scans with a 1 s interscan delay; Delay d4, 1.72 ms (¼J, J = 145 Hz); Delay d24, 0.86 ms (⅛J, J = 145 Hz); CT period d23, 14 ms. The total experiment time for a sample was 3.26 h. Processing to 2k × 2k datapoints (or just 1k × 1k for the plotted spectra) used fairly harsh Gaussian apodization (LB = −0.5, GB = 0.001) in F2 and Gaussian apodization (LB = –0.25, GB = 0.001) in F1 (without linear prediction).

Figure 2. Maize EL ^{1}H–^{13}C and ^{13}C–^{13}C correlation spectra. A) Partial HSQC spectrum from a 10%-^{13}C-enriched maize stover EL (to avoid the above issue of ^{13}C–^{13}C coupling). The spectrum reveals tricin (**T**, red), *trans-p*-coumarate (*t***C**, green), and (limited) *cis-p*-coumarate (*c***C**, cyan) correlations. The *p*-coumarate contours are broad in the proton dimension due to the structural diversity of components to which they are attached. All are free-phenolic entities acylating the γ-OH of lignin sidechains but may be associated with **G** or **S** units, and may be on *threo*- and *erythro*-isomers of β-ether units, phenylcoumarans, cinnamyl alcohol endgroups, and other more minor structures. Tricin units **T** are more structurally invariant, occurring as only 4′–O–β-linked units, but to both G and S moieties with *erythro*- or *threo*-stereochemistry. The spectrum was acquired with 618 increments (F1 acquisition time, 8.0 ms) of 16 scans for a total experiment time of 3.37 h. B) Partial HMBC spectrum from same sample as in A to help authenticate assignments. The spectrum was acquired with 308 increments (F1 acquisition time, 8.0 ms) of 128 scans per increment for a total experiment time of 14.56 h. C) Aromatic and ester carbonyl region from the C–C-FLOPSY spectrum of 100%-^{13}C-enriched maize straw lignin. The spectrum shows **T** (red), *t***C** (green), and *c***C** (cyan) correlations. Such ^{13}C–^{13}C correlation data from a fully-labeled lignin illustrate this experiment's value in mapping extensive carbon networks – all of tricin's protonated and quaternary carbons show informative and logical correlations. The dashed tricin assignment lines and the on-diagonal cross-marks are plotted from exact veratrylglycerol-(β–O–4′)-tricin ether model data, illustrating the excellent match with the lignin. Assignment lines for the *cis*- and *trans-p*-coumarates (*c***C** and *t***C**) are simply drawn through their contours. The spectrum was acquired with 1024 increments (F1 acquisition time, 13.2 ms) of 8 scans, with a 3 s interscan delay, for a total experiment time of 7.11 h.

Figure 2 Experimental Details

Figure 2A. Acquired from 11.66 to −0.66 ppm in F2 (^{1}H) with 3448 datapoints (acquisition time, 200 ms) and 215 to −5 ppm in F1 (^{13}C) with 618 increments (F1 acquisition time, 8.0 ms) of 16 scans with a 1 s interscan delay; Delay d4, 1.72 ms (¼J, J = 145 Hz); Delay d24, 0.89 ms (⅛J, J = 140 Hz). The total experiment time was 3.37 h. Processing to 2k × 2k datapoints used Gaussian apodization (LB = −0.5, GB = 0.001) in F2 and cosine-squared in F1 (without linear prediction).

Figure 2B. The HMBC was acquired from 11.66 to −0.66 ppm in F2 (^{1}H) with 4096 datapoints (acquisition time, 237.6 ms) and 215 to −5 ppm in F1 (^{13}C) with 308 increments (F1 acquisition time, 8.0 ms) of 128 scans with a 1 s interscan delay; Delay d2, 3.45 ms (½J, J = 145 Hz); Delay d6, 80 ms (½Jlr, J = 6.25 Hz). The total experiment time was 14.56 h. Processing to 4k × 2k datapoints (or just 2k × 1k for the plotted spectra) used matched Gaussian apodization (LB = −0.30, GB = 80/237.6 = 0.337) in F2 and sine-squared in F1 (with forward linear prediction with 32 coefficients).

Figure 2C. The ccflopsy16 pulseprogram, as listed in this SI, was used. Parameters: acquired from 214.25 to −4.25 ppm in F2 (^{13}C) with 4096 datapoints (acquisition time, 53.25 ms) and the same data-range in F1 (^{13}C) with 1024 increments (F1 acquisition time, 13.3 ms) of 8 scans with a 3 s interscan delay; the FLOPSY mixing time (d9) was 20 ms. The total experiment time was 7.11 h. Processing to 4k × 1k datapoints (or just 1k × 1k for the plotted spectrum) used Gaussian apodization (LB = −1, GB = 0.001) in F2 and Gaussian apodization (LB = −0.5, GB = 0.001) in F1 (without linear prediction).

Figure 3. Poplar stem EL. Aromatic and ester carbonyl region from the C–C-FLOPSY spectrum of 100%-^{13}C-enriched poplar stem lignin. The spectrum reveals the full set of *p*-hydroxybenzoate *p***HB** (purple) correlations. Again, there is sufficient dispersion in enough of the correlation peaks that, despite congestion from lignin peaks, reliable assignment of all carbons can be made, including for the quaternary *p***HB**$_{1}$, *p***HB**$_{4}$, and *p***HB**$_{7}$ carbons. The spectrum was acquired as for Figure 2C, with 8 scans per increment, for a total experiment time of 7.11 h.

Figure 3 Experimental Details

Figure 3. As for Figure 2C. Parameters: acquired from 214.25 to −4.25 ppm in F2 (^{13}C) with 4096 datapoints (acquisition time, 53.25 ms) and the same data-range in F1 (^{13}C) with 1024 increments (F1 acquisition time, 13.3 ms) of 8 scans with a 3 s interscan delay; the FLOPSY mixing time (d9) was 20 ms. The total experiment time was 7.11 h. Processing to 4k × 1k datapoints (or just 1k × 1k for the plotted spectra) used Gaussian apodization (LB = −1, GB = 0.001) in F2 and Gaussian apodization (LB = −0.5, GB = 0.001) in F1 (without linear prediction).

SUPPORTING EXPERIMENTS, DETAILS, AND FIGURES

Figure S1. Tricin T_8 HSQC peaks. In the past, pulse sequences designed to reduce ^{13}C–^{13}C coupling have improved resolution in 2D HSQC spectra of proteins,[17] but we have not had success with their implementation for lignins. Bruker's HSQC sequences designed for use with natural abundance compounds, "standard" or "normal" HSQC sequences, have a variety of issues when run on fully-^{13}C-labeled compounds. A couple of examples with 100%-^{13}C-labeled maize EL are shown here highlighting issues with ^{13}C–^{13}C coupling in ^{13}C-labeled materials. A) A modern, commonly used HSQC pulse sequence is sensitivity-improved and uses shaped pulses (Bruker pulseprogram hsqcetgpsisp2.2), and was used at 700 MHz to produce the spectra presented in Figure 1A and S1A using a typical set of parameters – see below. B) A slightly older but similar pulse sequence (sensitivity enhanced, and shaped pulses; Bruker pulseprogram hsqcetgpsisp.2) was run at 800 MHz for the same maize sample; it appears to show diminution of the ^{13}C coupling effect, but coupling is constant in Hz which is smaller in ppm at higher field strength. C) When using an older H–C HSQC sequence that does not include sensitivity improvement and shaped pulse elements (Bruker's pulseprogram hsqcetgp), we see reduction of the "multiplet" caused by ^{13}C-coupling. The sample is the same as in A and B, run at 800 MHz at higher-resolution in the ^{13}C indirect dimension (F1). This spectrum appears to reveal at least four tricin environments that may prove valuable for more detailed interpretation and assignment in the future. Tricin (**T**) units are anticipated to couple with canonical monolignols, both coniferyl and sinapyl alcohols, producing guaiacyl (**G**) and syringyl (**S**) β–O–4-tricin ethers as both *erythro* and *threo* isomers in each case, a likely explanation for the four NMR-distinct structures. Model compounds[25] for such structures will require meticulous examination in the appropriate solvent to ascertain the fidelity of this explanation.

Figure S1 Experimental Details

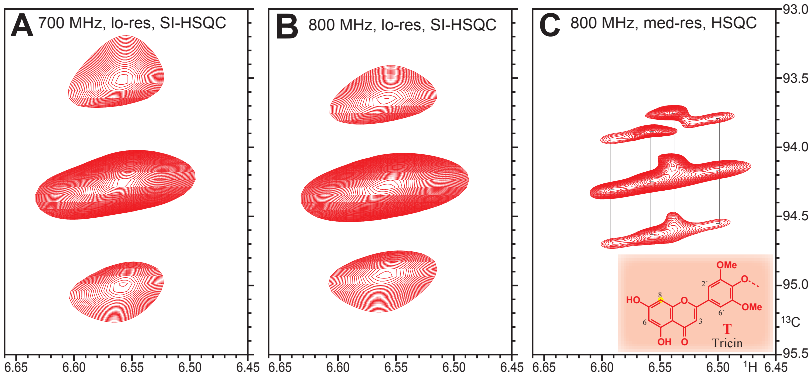

Figure S1A. 700 MHz, standard HSQC (Bruker pulseprogram hsqcetgpsisp2.2). Acquired from 11.66 to −0.66 ppm in F2 (^{1}H) with 3448 datapoints (acquisition time, 200 ms) and 215 to −5 ppm in F1 (^{13}C) with 1200 increments (F1 acquisition time, 15.5 ms) of 4 scans with a 1 s interscan delay; Delays d, 1.72 ms (¼J, J = 145 Hz); Delay d24, 0.89 ms (⅛J, J = 140 Hz). The total experiment time was 1.65 h. Processing to 4k × 4k datapoints used fairly harsh Gaussian apodization (LB = −0.5, GB = 0.001) in F2 and F1 (without linear prediction, 32 coefficients, in F1).

Figure S1B. 800 MHz, older HSQC (Bruker pulseprogram hsqcetgpsisp2). Acquired from 11.51 to −0.51 ppm in F2 (^{1}H) with 2048 datapoints (acquisition time, 106.5 ms) and 215 to −5 ppm in F1 (^{13}C) with 1024 increments (F1 acquisition time, 11.6 ms) of 4 scans with a 1 s interscan delay; Delay d4, 1.72 ms (¼J, J = 145 Hz); Delay d24, 0.60 ms (⅛J, J = 208 Hz). The total experiment time was 1.29 h. Processing to 4k × 4k datapoints used fairly harsh Gaussian apodization (LB = −0.5, GB = 0.001) in F2 and Gaussian apodization (LB = –0.2, GB = 0.001) in F1 (without linear prediction).

Figure S1C. 800 MHz, older HSQC (Bruker pulseprogram hsqcetgp) at higher resolution in F1. Acquired from 10.70 to −1.30 ppm in F2 (^{1}H) with 2048 datapoints (acquisition time, 106.5 ms) and 215 to −5 ppm in F1 (^{13}C) with 2560 increments (F1 acquisition time, 28.9 ms) of 16 scans with a 2 s interscan delay; Delay d4, 1.56 ms (¼J, J = 160 Hz). The total experiment time was 24.24 h. Processing to 4k × 16k datapoints used Gaussian apodization (LB = −0.5, GB = 0.01) in F2 and exponential multiplication (LB = 1) in F1 (with linear prediction, 32 coefficients).

Supporting Information: Profiling ferulates via a fully-^{13}C-labeled sorghum leaf CW sample

Spectra of fully-^{13}C-labeled sorghum leaf CW samples enable the previously unassigned *cis*-ferulate (*c***F**) peaks to also be elucidated (Figure S2), thereby validating recent speculation regarding their likely presence.[28] The assignments can be confidently ascribed by comparison with the normal HSQC spectrum from an older sample of unlabeled **FA-Ara** (Figure S2A), 5-*O*-feruloyl-1-*O*-methyl-arabinofuranoside, an excellent model compound for ferulates on arabinoxylans in grasses.[42,65] We are grateful to Richard F. Helm for synthesis of the original **FA-Ara**.[42] A low level (~5%) of isomerization to the *cis*-ferulate had occurred in this sample, along with some cyclodimerization to the cyclic dicarboxylic acid, truxillic acid **FF**, an also well-established photochemical reaction common to ferulates.[66]

All of the *c***F** peaks except the overlapping *c***F**$_5$ peak are readily identified in the two CT-HSQC spectra from the sorghum leaf CW sample (Figures S2B and S2C). The photochemically derived cyclodimer **FF** is essentially undetectable suggesting that such dimerization is less prevalent when the ferulates that need to encounter each other are tethered to full arabinoxylan chains.

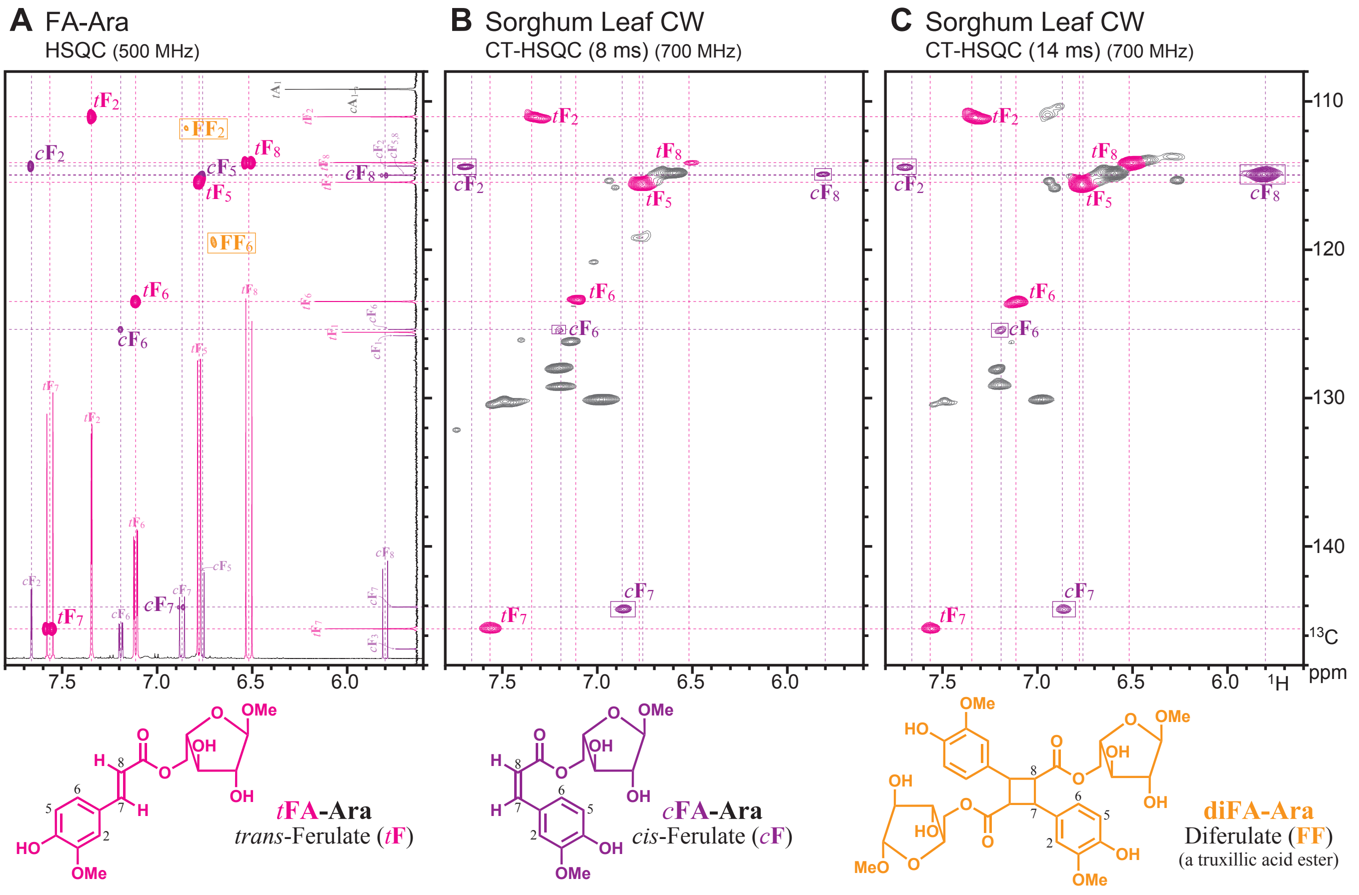

Figure S2. Profiling ferulate units in sorghum leaf CW samples. A) HSQC (at 500 MHz) of unlabeled **FA-Ara** (5-*O*-feruloyl-1-*O*-methyl-arabinofuranoside),[42] a model for ferulate on arabinoxylan polysaccharides common to grasses. The sample was UV-irradiated at 250 nm in MeOH for 1 h to produce higher levels (~21%) of *cis*-ferulates *c***F** and the cyclodimer **FF**, and then purified via thick-layer chromatography to isolate the mixture of *cis*- and *trans*-ferulates for NMR here. The $\mathbf{F}_7$, $\mathbf{F}_8$, $\mathbf{F}_2$, and $\mathbf{F}_6$ ^{1}H–^{13}C correlation peaks are particularly disparate between the two isomers, but the partially-overlapped $c\mathbf{F}_5$ peak is likely also discernable. The original unpurified material with low levels (~5%) of *c***F** also contained the cyclodimer **FF**. Its NMR, from the tlc-separated product, displayed characteristic correlations at 4.22/40.7 ($\mathbf{FF}_7$), 3.87/46.3 ($\mathbf{FF}_8$); other peaks were 6.85/111.6 ($\mathbf{FF}_2$), 6.70/119.5 ($\mathbf{FF}_6$), 6.70/115.3 ($\mathbf{FF}_5$), 3.76/55.6 ($\mathbf{FF}_{3\text{-OMe}}$). The $\mathbf{FF}_6$ and $\mathbf{FF}_2$ peaks from this cyclodimer, shown as overlays in orange boxes, are from a spectrum of the separated product. Characteristic correlations at 4.22/40.7 ($\mathbf{FF}_7$), 3.87/46.3 ($\mathbf{FF}_8$) are readily seen in the aliphatic region of the spectrum (not shown). B-C) Partial CT-HSQC spectra (aromatic/double-bond region, 700 MHz) with B) 8 ms and C) 14 ms constant-time periods from sorghum leaf CW material. Each displays the clearly delineated *trans*- and *cis*-ferulates (*t***F** and *c***F**), with the latter revealing most of the analogous correlations at a 16-fold lower contour level as purple-colored contours in purple boxes. The assignment lines in B-C are not drawn through the centers of the correlations, but are directly from the FA-Ara spectrum in A to illustrate the acceptable match. The minor differences are easily attributable to the difference in the 1-*O*-substitution, a simple methyl in the **FA-Ara** model (Figure S2A) *vs* a xylan unit in the CW (Figures S2B-C).

Figure S2 Experimental Details

Figure S2A. Acquired on a Bruker Biospin AVANCE 500 MHz spectrometer fitted with a 5-mm TCI (triple resonance; ^{1}H, ^{13}C, ^{15}N) gradient cryoprobe with inverse geometry, from 10 to 0 ppm in F2 (^{1}H) with 1998 datapoints (acquisition time, 200 ms) and 200 to 0 ppm in F1 (^{13}C) with 400 increments (F1 acquisition time, 7.95 ms) of 16 scans with a 1 s interscan delay; Delay d4, 1.72 ms (¼J, J = 145 Hz); Delay d24, 0.89 ms (⅛J, J = 140 Hz). The total experiment time was 2.19 h. Processing to 2k × 2k datapoints used Gaussian apodization (LB = −0.05, GB = 0.001) in F2 and Gaussian apodization (LB = –0.20, GB = 0.001) in F1 (without linear prediction). The $\mathbf{FF}_6$ and $\mathbf{FF}_2$ peaks from the cyclodimer, shown as overlays in orange boxes, are from a separate spectrum of the separated product.

Figure S2B. This plot was from the same spectrum as in Figure 1E – parameters as therein. Correlations for the *c***F** peaks are at a 16-fold lower contour level as purple-colored contours in purple boxes.

Figure S2C. This plot was from the same spectrum as in Figure 1F – parameters as therein. Correlations for the *c***F** peaks are at a 16-fold lower contour level as purple-colored contours in purple boxes.

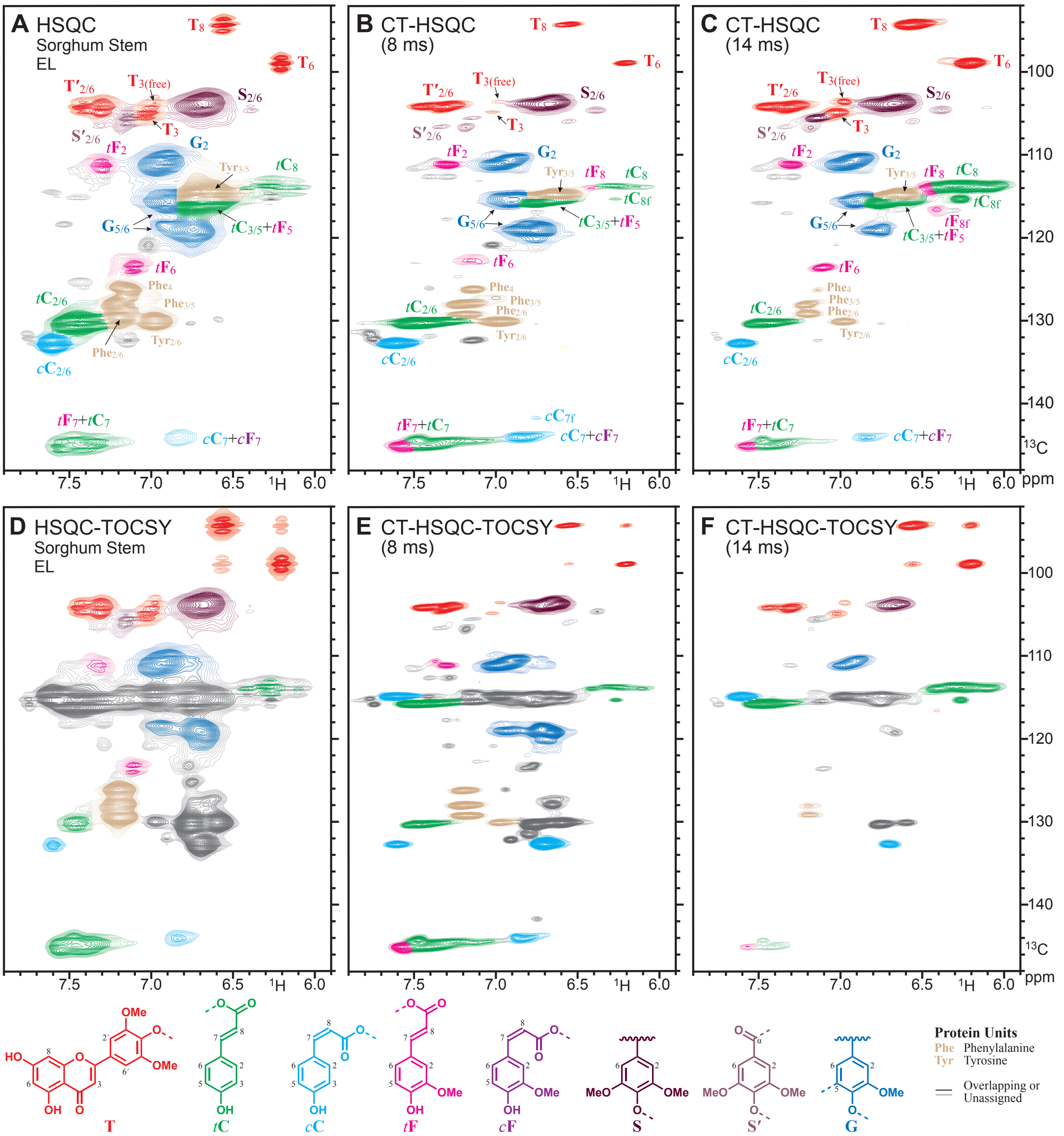

Figure S3. Sorghum Stem EL ^{1}H–^{13}C correlation spectra. The top row (A-C) is analogous to that in Figure 1 in the main paper from sorghum stem lignin, and using the same dataset, but showing a truncated proton chemical shift range for additional clarity (at the expense of being able to see the cC$_8$ peaks). A) The aromatic region of a normal HSQC spectrum of fully ^{13}C-labeled sorghum stem lignin (EL) illustrates the challenges arising from ^{13}C–^{13}C coupling, as illustrated particularly by tricin's **T**$_6$ and **T**$_8$ correlations in the upper-right of the spectrum. The apparent triplet for these peaks is greater than expected from just the ~45 Hz ^{13}C–^{13}C couplings due to their evolution during the gradients used for coherence selection,[50] as explained for Figure S1. The spectrum was acquired with 4 scans per increment, and had a total acquisition time of 1.65 h. B) A constant-time HSQC (CT-HSQC) pulse sequence mitigates the issues with ^{13}C–^{13}C coupling producing, in the carbon dimension, sharp **T**$_6$ and **T**$_8$ peaks, for example. The intensities depend on the constant-time period (CT = n/J$_{C\text{–}C}$). C) As for B but with a CT period of 14 ms, showing the superior sensitivity for the tricin peaks relative to the normal syringyl lignin **S**$_{2/6}$ peak. The spectrum was acquired with 8 scans per increment, with a total acquisition time of 3.26 h. D-F) Analogous CT-HSQC-TOCSY spectra, all with a TOCSY mixing times of 60 ms. These spectra nicely show the correlation of the **T**$_6$ and **T**$_8$ peaks, for example, of quite low intensity due to the small (~2 Hz) ^{1}H–^{1}H coupling constant between the protons. Again, the CT-HSQC experiments (E and F) mitigate the issues with ^{13}C–^{13}C coupling producing in the carbon dimension sharp **T**$_6$ and **T**$_8$ peaks (and their TOCSY-correlation peaks), for example. Other correlation peaks severely overlap rendering it challenging to color the correlations based on their structural assignments. However, unlike in the HSQCs of Figure 1, we made some attempt to color-delineate the various contributions in the HSQC experiments (A-C), not with complete fidelity, that were not attempted in Figure 1, but this was not viable for the HSQC-TOCSY spectra (D-F).

Figure S3 Experimental Details

Figure S3A. Parameters are the same as for Figure 1A.

Figure S3B. Parameters are the same as for Figure 1B.

Figure S3C. Parameters are the same as for Figure 1C.

Figure S3D. The normal HSQC-TOCSY (Bruker standard pulse sequence hsqcdietgpsisp.2) experiments on ELs used the same acquisition and processing parameters as for the HSQC above but with a TOCSY mixing time (d9) of 60 ms. Acquired from 11.66 to −0.66 ppm in F2 (^{1}H) with 3448 datapoints (acquisition time, 200 ms) and 215 to −5 ppm in F1 (^{13}C) with 1200 increments (F1 acquisition time, 15.5 ms) of 4 scans with a 1 s interscan delay; Delay d4, 1.72 ms (¼J, J = 145 Hz); Delay d24, 0.89 ms (⅛J, J = 140 Hz); TOCSY mixing time d9 = 60 ms. The total experiment time for a sample was 1.74 h. Processing to 2k × 2k datapoints (or just 1k × 1k for the plotted spectra) used Gaussian apodization (LB = −0.5, GB = 0.001) in F2 and Gaussian apodization (LB = –0.25, GB = 0.001) in F1 (without linear prediction).

Figure S3E. The CT-HSQC-TOCSY experiments used the hsqcdictetgpsisp.mt2 pulseprogram as listed in the last section of this SI. The parameters were the same as for the above CT-HSQC run in Figure S3B and Figure 1B but with a TOCSY mixing time (d9) of 60 ms. The resolution in F1 was limited depending on the constant time (CT) period. Acquired from 11.66 to −0.66 ppm in F2 (^{1}H) with 3448 datapoints (acquisition time, 200 ms) and 215 to −5 ppm in F1 (^{13}C) with 1052 increments (F1 acquisition time, 13.6 ms) of 8 scans with a 1 s interscan delay; Delay d4, 1.72 ms (¼J, J = 145 Hz); Delay d24, 0.86 ms (⅛J, J = 145 Hz); TOCSY mixing time d9 = 60 ms; CT period d23, 8 ms. The total experiment time was 3.02 h. Processing to 2k × 2k datapoints (or just 1k × 1k for the plotted spectra) used Gaussian apodization (LB = −0.5, GB = 0.001) in F2 and Gaussian apodization (LB = –0.25, GB = 0.001) in F1 (without linear prediction).

Figure S3F. The parameters were the same as for the above CT-HSQC run in Figure S3C and Figure 1C but with a TOCSY mixing time (d9) of 60 ms. Acquired from 11.66 to −0.66 ppm in F2 (^{1}H) with 3448 datapoints (acquisition time, 200 ms) and 215 to −5 ppm in F1 (^{13}C) with 1176 increments (F1 acquisition time, 15.2 ms) of 8 scans with a 1 s interscan delay; Delay d4, 1.72 ms (¼J, J = 145 Hz); Delay d24, 0.86 ms (⅛J, J = 145 Hz); TOCSY mixing time d9 = 60 ms; CT period d23, 14 ms. The total experiment time was 3.41 h. Processing to 2k × 2k datapoints (or just 1k × 1k for the plotted spectra) used fairly harsh Gaussian apodization (LB = −0.5, GB = 0.001) in F2 and Gaussian apodization (LB = –0.25, GB = 0.001) in F1 (without linear prediction).

Supporting Information: HSQC-TOCSY experiments; CT-HSQC-TOCSY

The ^{1}H–^{13}C HSQC-TOCSY experiment combines experiments to provide through-bond correlations between a ^{13}C-attached proton and all other coupled protons, *i.e.*, within a proton-coupling network.[67] In the typical situation, each proton correlates with each carbon and each carbon correlates with each proton in the coupling network. The beautiful redundancy of information makes identifying components within the same molecular structure quite trivial. Scant mention of CT variants is made in the literature. Although the experiment doesn't appear in the Bruker pulseprogram library, other researchers appear to have implemented the experiment but few details can be found about its effectiveness or value.[68] As for the HSQC part of the 2D HSQC-TOCSY experiment (*e.g.*, utilizing sensitivity enhancement and DIPSI2 for homonuclear Hartman-Hahn mixing, Bruker pulseprogram hsqcdietgpsisp.2), the experiment encounters similar challenges arising from ^{13}C–^{13}C coupling when applied to fully-labeled samples (Figure S3D). To address these issues, we have implemented a constant-time analog, CT-HSQC-TOCSY (pulseprogram hsqcdictetgpsisp.mt, as detailed in the last section of this SI). The various HSQC and HSQC-TOCSY spectra (Figure S3) of the same sorghum stem EL sample as used for Figures 1A-C, also with CT periods of 8 or 14 ms to again alleviate the ^{13}C–^{13}C coupling issues, effectively reveal even the weak (due to the 2 Hz ^{1}H–^{1}H coupling constant between the protons) correlations between tricin's $\mathbf{T}_6$ and $\mathbf{T}_8$ peaks. This confirms their existence within the same proton coupling network. In our experience, and with these polymeric samples, this CT-HSQC-TOCSY experiment appears to be somewhat less valuable for revealing coupling networks than the standard HSQC-TOCSY experiment on natural-abundance or 10%-^{13}C-enriched biomass.[1,4]

Supporting Information: Short explanation of terms: spinlock time, TOCSY or FLOPSY mixing time

The TOCSY experiment (TOtal COrrelation SpectroscopY) gets its name from the result – correlation between connected spins (3 or fewer bonds). It obtains this through a spin-lock by basically setting to zero the chemical shift difference, leaving only the full scalar coupling as the Hamiltonian. So "spin-lock" or "spinlock" is the term to describe how one obtains total correlation spectra. There are different methods to implement the spin-lock, including DIPSI and FLOPSY, in which the pulse lengths and phases set the magnetization to traverse the sphere to obtain this spinlock. In the case of FLOPSY, it was computer-optimized by solving the Bloch equations. As a result, the FLOPSY mixing time is a spinlock time for which the B1 field is used for the FLOPSY pulse train.

Supporting Information References (additional to those in the main text)

The following references pertain only to the SI; others are number as in the main paper.

65. Lin, S.; Agger, J. W.; Wilkens, C.; Meyer, A. S., Feruloylated arabinoxylan and oligosaccharides: Chemistry, nutritional functions, and options for enzymatic modification. *Annual Review of Food Science and Technology* **2021,** *12*, 331–354. [DOI: 10.1146/annurev-food-032818-121443]
66. Hartley, R. D.; Morrison, W. H., III; Himmelsbach, D. S.; Borneman, W. S., Cross-linking of cell wall phenolic arabinoxylans in graminaceous plants. *Phytochemistry* **1990,** *29* (12), 3705–3709. [DOI: 10.1016/0031-9422(90)85317-9]
67. Willker, W.; Leibfritz, D.; Kerssebaum, R.; Bermel, W., Gradient selection in inverse heteronuclear correlation spectroscopy. *Magnetic Resonance in Chemistry* **1992,** *31*, 287–292. [DOI: 10.1002/mrc.1260310315]
68. Aguilar, F.; Banaei, N.; Zhang, Y., ^{1}H, ^{13}C and ^{15}N resonance assignments and structure prediction of translation initiation factor 1 from *Clostridium difficile*. *Biomolecular NMR Assignments* **2019,** *13* (1), 91–95. [DOI: 10.1007/s12104-018-9858-8]

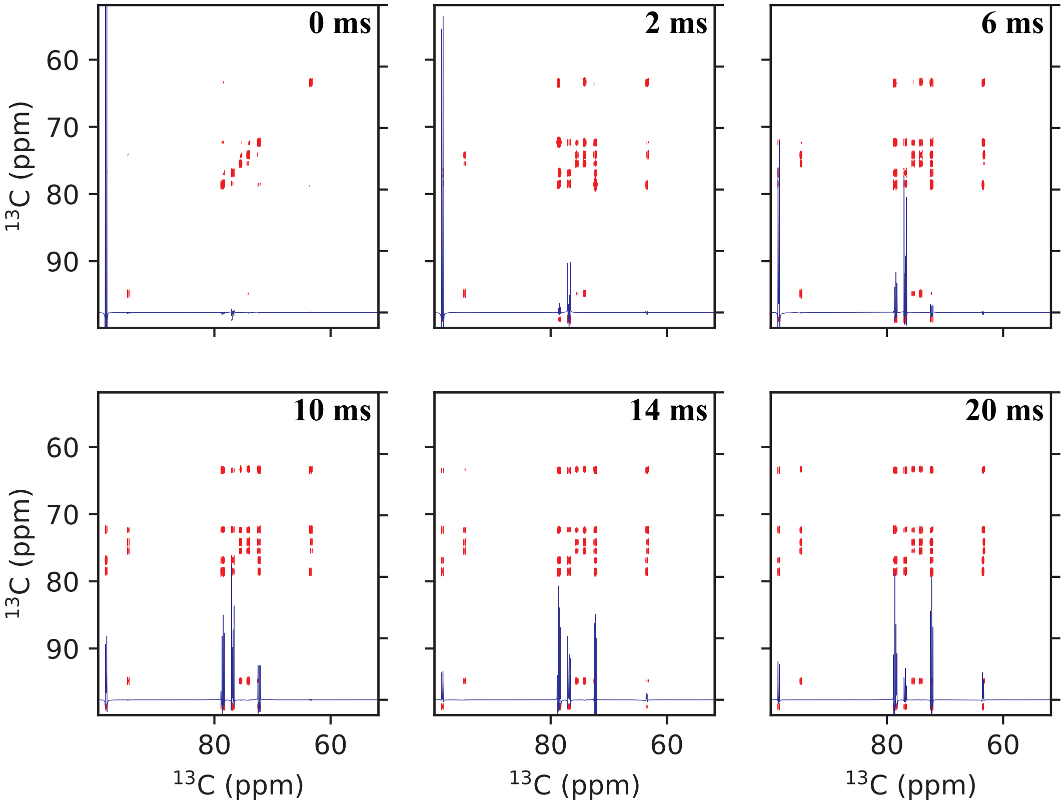

Figure S4. C–C-FLOPSY experiments on fully-^{13}C-labeled glucose. Spectra were recorded on a 5 mM sample in 550 µL of D_2O at 800 MHz, with a ^{13}C window of 62 ppm centered at 80 ppm in both dimensions, sampling 2k points in the direct acquisition dimension and 256 points in the indirect dimension, with 4 scans per increment, and a d1 of 2 s; the total acquisition time was 38 min per spectrum. The FLOPSY mixing time was varied from 0 to 20 ms, with a 12.5 kHz ^{13}C B_1 field. In blue for each is shown the extracted 1D spectrum at the chemical shift of the anomeric carbon (98.7 ppm).

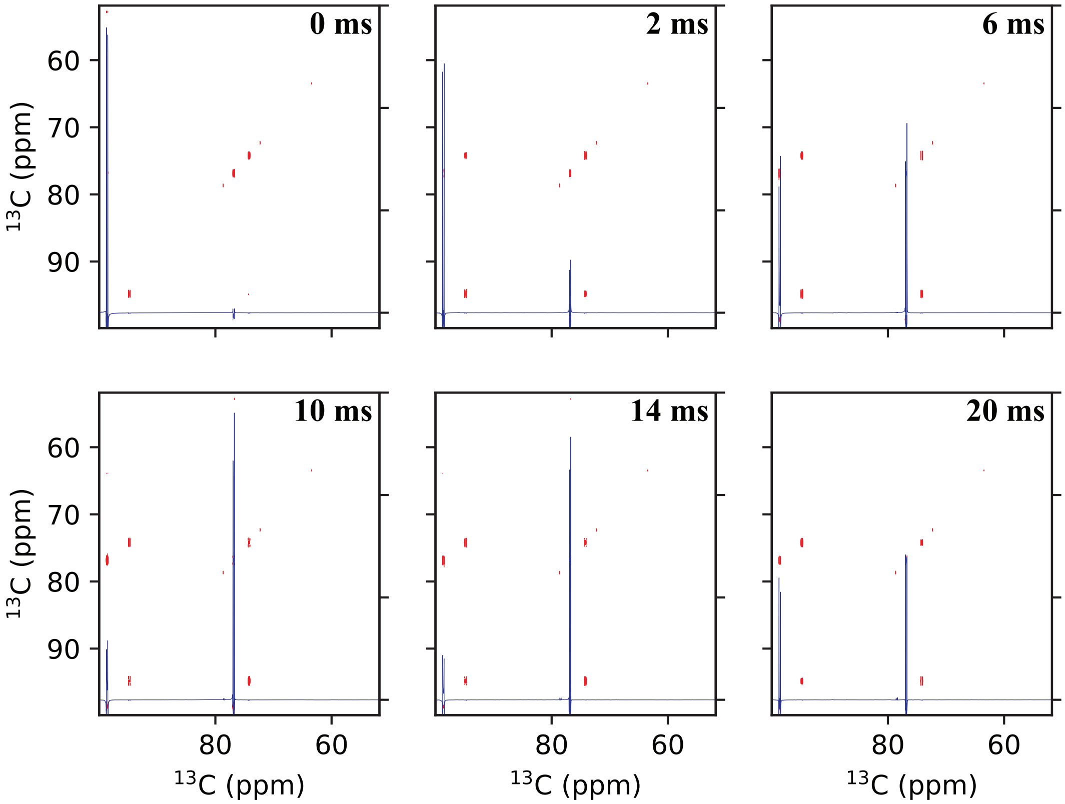

Figure S5. C–C-FLOPSY experiments on 1,2-di-^{13}C-labeled glucose. Spectra were recorded on a 5 mM sample in 550 µL of D_2O at 800 MHz using the same parameters as for Figure S4. In blue for each is shown the extracted 1D spectrum at the chemical shift of the anomeric carbon (98.7 ppm).

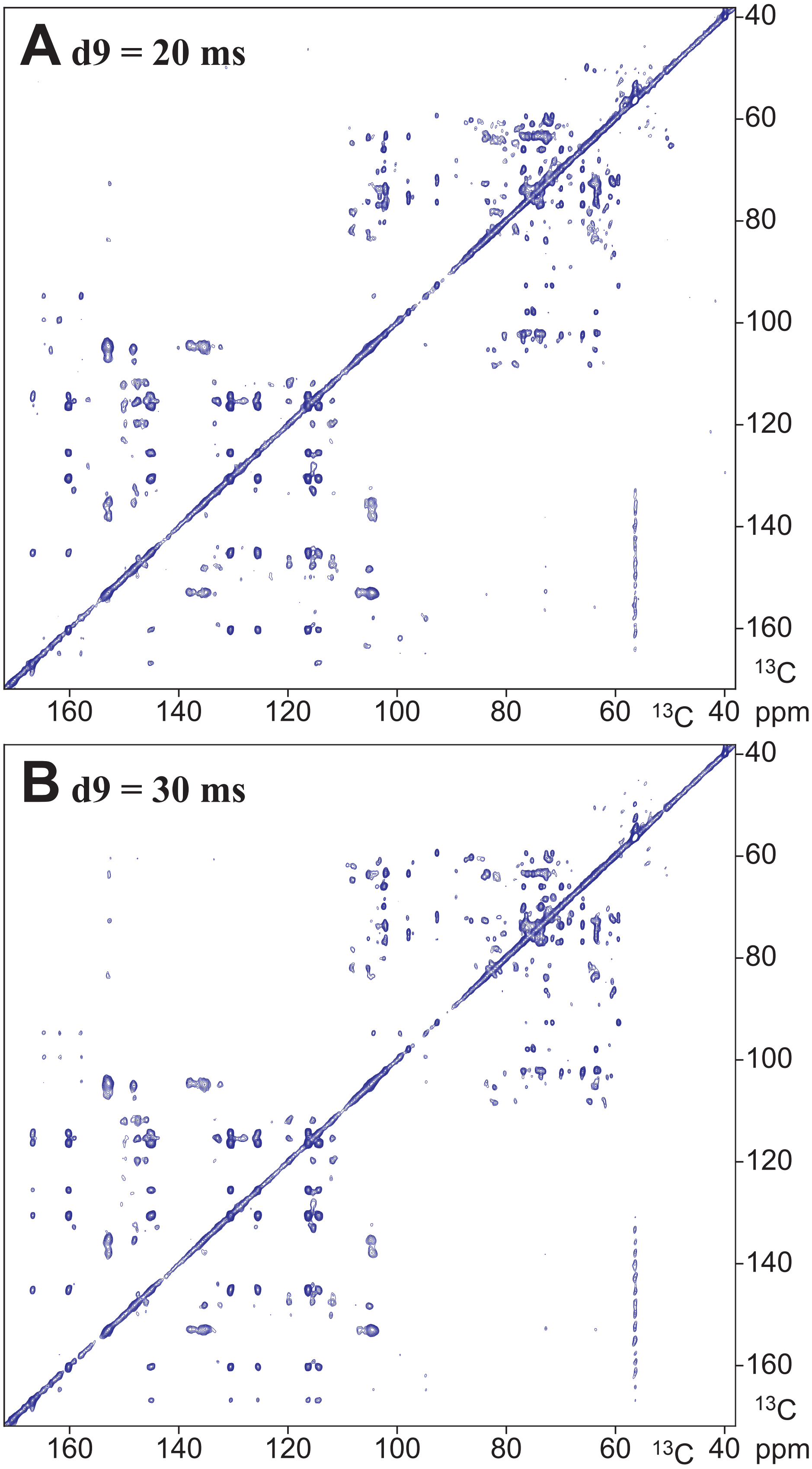

Figure S6. C–C-FLOPSY experiments at 900 MHz on the fully-^{13}C-labeled maize EL. Comparison of mixing times d9 (FLOPSY mixing time) of 20 ms *vs* 30 ms, each with a p6 [f1 channel (^{13}C) 90° low-power pulse] of 20 µs. The maize EL sample in DMSO-d_6 is the same as that used for the spectra in Figure 1, but run at 328 K. A) 20 ms. B) 30 ms. Note that d9 = 30 ms was pushing the cryoprobe to its limit – the specifications for the cryoprobe are 40 ms of p6 = 22 µs; at 30 ms with a 20 µs pulse, the probe was on the brink of warming up. As noted in the main paper, we find that a d9 of 20 ms provides excellent C–C relaying under safe NMR conditions.

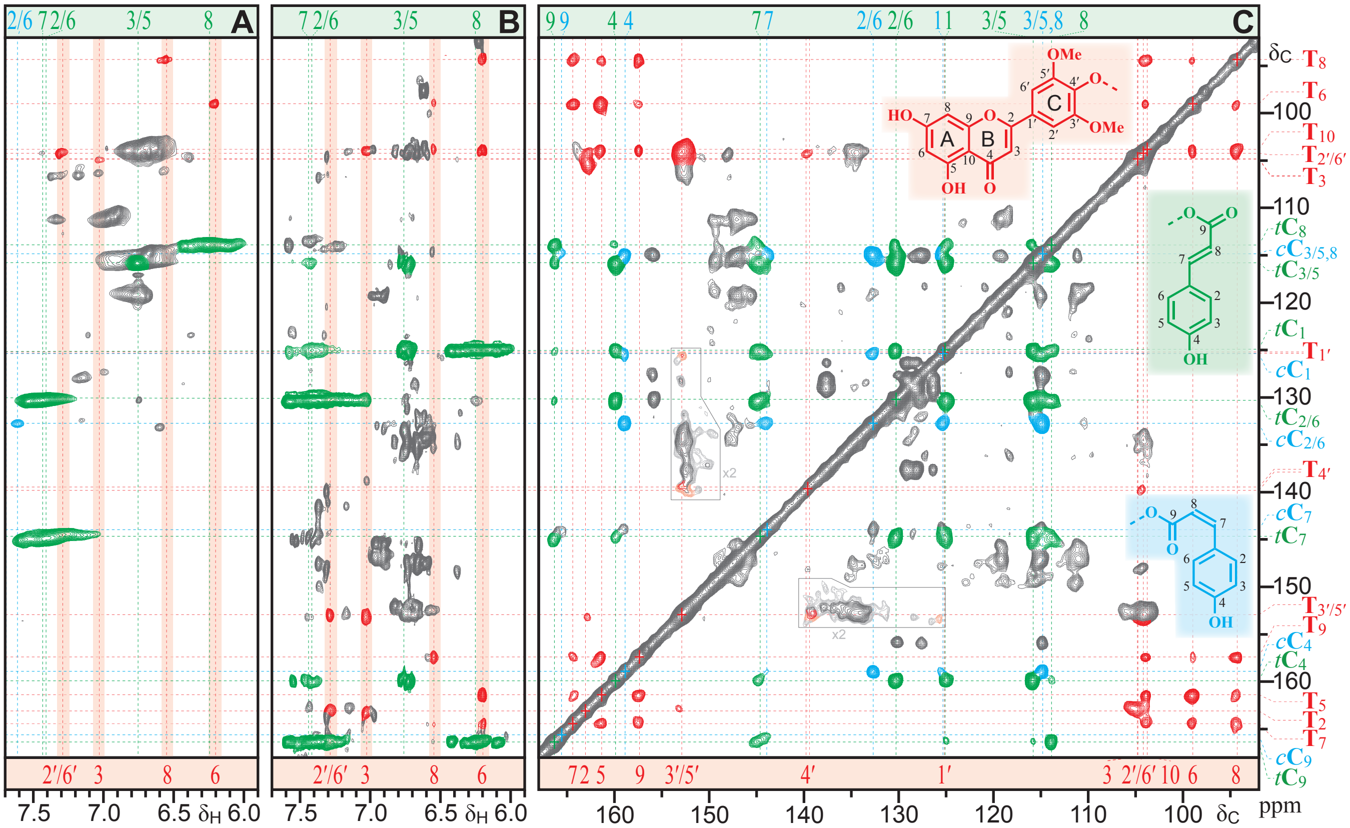

Figure S7. Maize EL ^{1}H–^{13}C correlation spectra and sorghum stem EL C–C-FLOPSY spectrum. This figure is analogous to Figure 2 in the main paper but Figure S7C is the C–C-FLOPSY spectrum derived from 100%-^{13}C-labeled sorghum stem EL (instead of maize EL in Figure 2C). Neither an unlabeled sorghum control nor 10%-^{13}C-labeled sorghum were available to obtain a comparable HMBC. A-B) As the lignins and pendant units are similar across monocots, the maize analogs are plotted here for reference. C) The plot is on the same scale as Figure 2C for comparison. The boxed regions show a 2× expansion of those regions to identify the weaker **T** correlations. Of particular note is that many of the tricin (**T**) peaks are even more prevalent in this sorghum spectrum than in the maize spectrum of Figure 2C. As for Figure 2, the dashed tricin assignment lines and the on-diagonal cross-marks may appear as if they were simply drawn through the various contours, but these are actually plotted from exact veratrylglycerol-(β–O–4′)-tricin ether model data, clearly matching the lignin. Assignment lines and diagonal cross-marks for the *cis*- and *trans*-*p*-coumarates (*c***C** and *t***C**) are drawn to exactly match those from maize EL in Figure 2, again illustrating the excellent match between monocot samples.

Figure S7 Experimental Details

Figure S7A. Spectrum and parameters are from maize, for comparison, as for Figure 2A.

Figure S7B. Spectrum and parameters are as for Figure 2B.

Figure S7C. Sorghum stem EL C–C-FLOPSY parameters are the same as for Figure 2C except 32 scans were acquired; 8 scans were sufficient to provide excellent data. Parameters: acquired from 214.25 to −4.25 ppm in F2 (^{13}C) with 4096 datapoints (acquisition time, 53.25 ms) and the same data-range in F1 (^{13}C) with 1024 increments (F1 acquisition time, 13.3 ms) of 32 scans with a 3 s interscan delay; the FLOPSY mixing time (d9) was 20 ms. The total acquisition time was 28.35 h. Processing to 4k × 1k datapoints (or just 1k × 1k for the plotted spectra) used Gaussian apodization (LB = −0.5, GB = 0.001) in F2 and F1 (without linear prediction).

Figure S8. Sorghum leaf EL vs leaf CW C–C-FLOPSY spectra. A) Analog of Figure 2C and Figure S7C but for C–C-FLOPSY data from 100%-^{13}C-labeled EL from sorghum leaves, showing the higher level of tricin **T** and the lower level of *p*-coumarates **C**. B) An analogous plot for sorghum leaf 100%-^{13}C-enriched CW material illustrating how the C–C-FLOPSY experiment is valuable even on unfractionated ball-milled biomass that has been simply swollen in DMSO. Despite variations in cross-peak intensities and the greater congestion in the CW spectrum (Figure S8B), the range of resolved correlations remains comparable to those obtained from the more laboriously isolated ELs. Plotted on the same scale as for Figures 2C and S7C for comparison, the dashed tricin assignment lines are plotted from exact veratrylglycerol-(β–O–4′)-tricin ether model data, clearly matching the lignin. Assignment lines and diagonal cross-marks for the *cis*- and *trans*-*p*-coumarates (*c***C** and *t***C**) are drawn to exactly match those from maize EL in Figure 2, again illustrating the excellent match between monocot samples.

Figure S8 Experimental Details

Figure S8A. Sorghum Leaf EL C–C-FLOPSY parameters are the same as for Figure 2C. Parameters: acquired from 214.25 to −4.25 ppm in F2 (^{13}C) with 4096 datapoints (acquisition time, 53.25 ms) and the same data-range in F1 (^{13}C) with 1024 increments (F1 acquisition time, 13.3 ms) of 8 scans with a 3 s interscan delay; the FLOPSY mixing time (d9) was 20 ms. The total experiment time was 7.11 h. Processing to 4k × 1k datapoints (or just 1k × 1k for the plotted spectra) used Gaussian apodization (LB = −0.5, GB = 0.001) in F2 and F1 (without linear prediction).

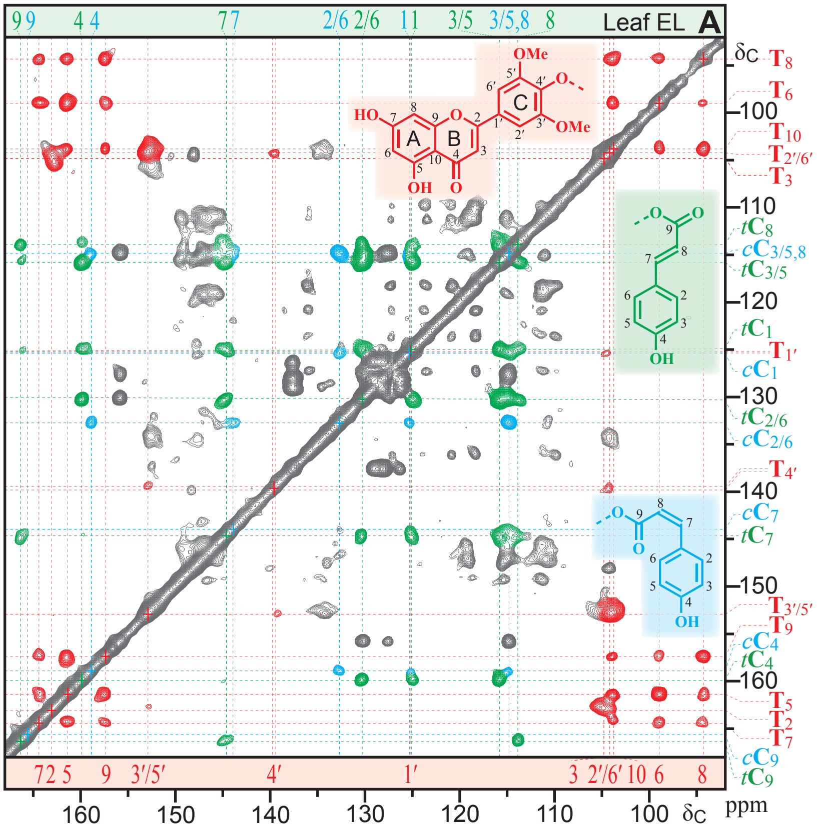

Figure S8B. Sorghum Leaf CW C–C-FLOPSY parameters are the same as for Figure S8A except 96 scans were acquired per increment. Parameters: acquired from 214.25 to −4.25 ppm in F2 (^{13}C) with 4096 datapoints (acquisition time, 53.25 ms) and the same data-range in F1 (^{13}C) with 1024 increments (F1 acquisition time, 13.3 ms) of 96 scans with a 1 s (instead of the usual 3 s) interscan delay; the FLOPSY mixing time (d9) was 20 ms. The total experiment time was 30.36 h. [Note that 96 scans was excessive and suitable spectra could be acquired using the same parameters as for Figure 8A, with 8 scans and a 3 s interscan delay in 7.11 h. Also note that a 1 s interscan delay is reasonable for these CW samples, considerably reducing the total experiment time]. Processing to 4k × 1k datapoints (or just 1k × 1k for the plotted spectra) used Gaussian apodization (LB = −1, GB = 0.001) in F2 and Gaussian apodization (LB = –0.5, GB = 0.001) in F1 (without linear prediction).

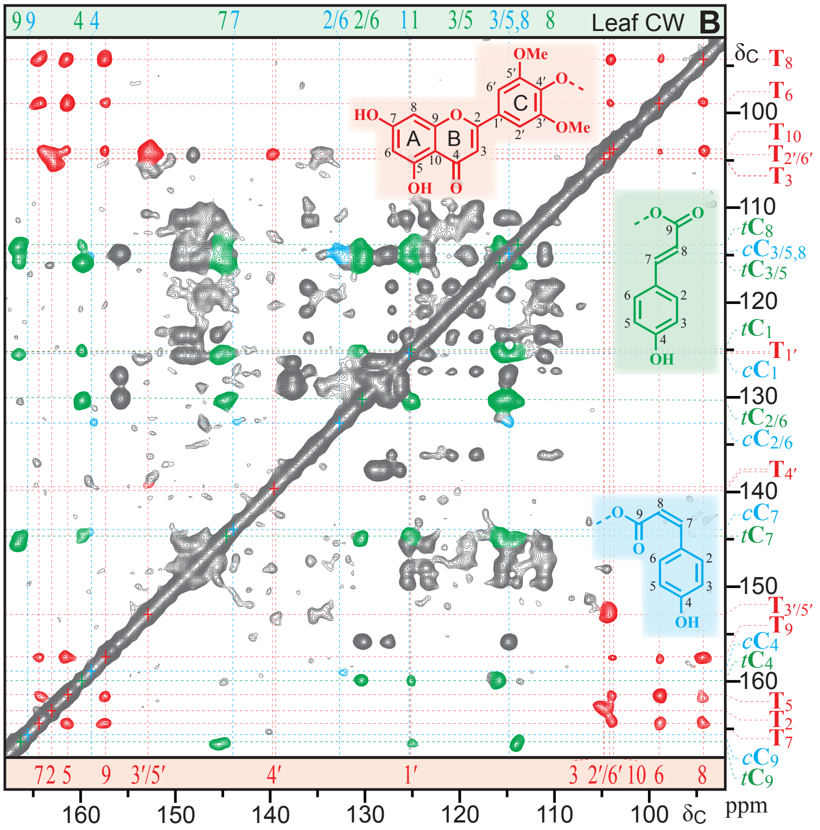

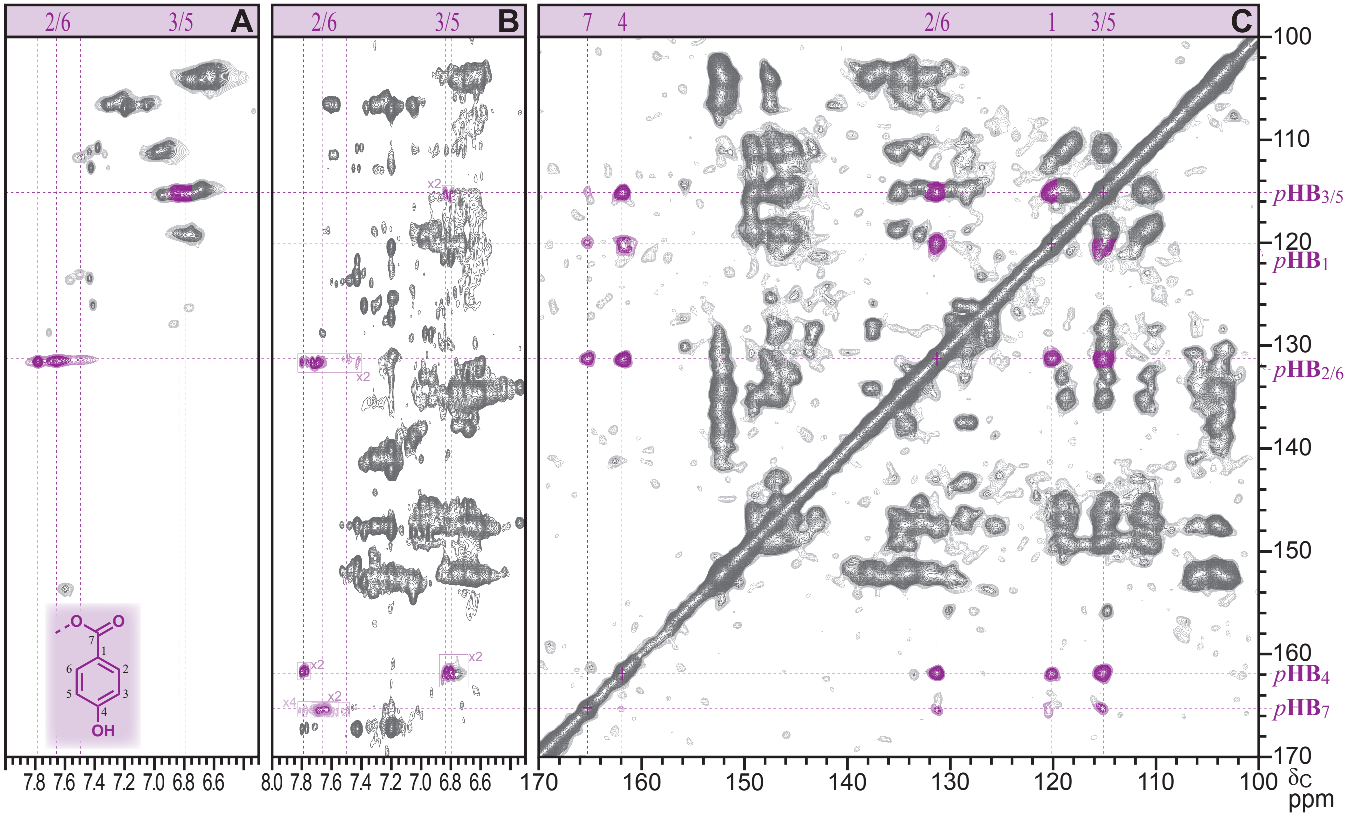

Figure S9. Poplar lignin spectra. A) Partial HSQC spectrum from a natural aspen (*Populus tremuloides*) milled wood lignin (MWL) showing the limited *p***HB** correlations. Partially-labeled poplar materials were not available. The *p*-hydroxybenzoate contours are broad in the proton dimension due to the structural diversity of components to which they are attached. All are free-phenolic entities acylating the γ-OH of lignin sidechains but may be associated with **G** or **S** units, and may be on *threo*- and *erythro*-isomers of β-ether units, phenylcoumarans, cinnamyl alcohol endgroups, and other more minor structures. B) Partial HMBC spectrum from the same sample as in A to help authenticate some assignments. C) Aromatic and ester carbonyl region from the C–C-FLOPSY spectrum of 100%-^{13}C-enriched poplar stem lignin, from the same experimental data as for Figure 3. The spectrum reveals the full set of *p*-hydroxybenzoate *p***HB** (purple) correlations. Again, there is sufficient dispersion in enough of the correlation peaks that, despite congestion from lignin peaks, reliable assignment of all carbons can be made, including for the quaternary *p***HB**$_1$, *p***HB**$_4$, and *p***HB**$_7$ carbons.

Note that auxiliary spectra, as for Figure 2 with maize, are less satisfying for poplar lignins, in part because of the paucity of protonated carbons in the pendent *p***HB** units – there are only two (sets of) protonated carbons, *p***HB**$_{2/6}$ and *p***HB**$_{3/5}$. In part because of the variety of proton *p***HB**$_{2/6}$ shifts, HMBC correlations from unlabeled EL or CW samples, acquired over many years on many different samples, failed to reveal useful correlations except for protons *p***HB**$_{2/6}$ to carbons *p***HB**$_4$ and perhaps *p***HB**$_7$. There are also strong apparent correlation peaks from protons *p***HB**$_{2/6}$ to carbons *p***HB**$_{2/6}$ because protons *p***HB**$_2$ can correlate long-range with carbons *p***HB**$_6$ and *vice versa*. The two-bond correlation to *p***HB**$_1$ is simply not evident under a variety of acquisition and processing regimes. The two-bond correlation to *p***HB**$_{3/5,}$ if present, is masked by other lignin peaks in this region. These difficulties render all the more impressive the ease with which the entire carbon network can be so elegantly traced out via the C–C-FLOPSY experiment on the fully-labeled sample, with all 5 of the non-equivalent carbons correlating with the other four carbons in the *p***HB** unit (Figures 3, S9C).

Figure S9 Experimental Details

Figure S9A. Acquired on a Bruker BioSpin AVANCE III 500 MHz NMR spectrometer fitted with a 5 mm, z-gradient Prodigy TCI cryoprobe at 298 K. The HSQC experiment used the Bruker standard pulse program hsqcetgpsisp2.2, acquired from 11.88 to 0.12 ppm in F2 (^{1}H) with 1024 datapoints (acquisition time, 87 ms) and 212.5 to -2.5 ppm in F1 (^{13}C) with 512 increments (F1 acquisition time, 9.47 ms) of 25 scans with a 1 s interscan delay; Delay d4, 1.72 ms ($^1\!/_{4J}$, J = 145 Hz); Delay d24, 0.89 ms ($^1\!/_{8J}$, J = 140 Hz). The total experiment time was 3.97 h. Processing to 2k × 2k datapoints used Gaussian apodization (LB = −0.2, GB = 0.001) in F2 and cosine-bell squared in F1 (without linear prediction). The central DMSO solvent peak was used as the internal reference (δ_C 39.5, δ_H 2.49 ppm) in 1D spectra and the reference values (SR in Bruker TopSpin) applied to the 2D spectra.

Figure S9B. Acquired on the same instrument as for Figure S9A. The HMBC used the standard Bruker hmbcetgpl3nd pulse program, acquired from 11.88 to 0.12 ppm in F2 (^{1}H) with 2048 datapoints (acquisition time, 174 ms) and 212.5 to -2.5 ppm in F1 (^{13}C) with 1024 increments (F1 acquisition time, 18.94 ms) of 60 scans with 30% NUS sampling density and a 1 s interscan delay; Delays were set for $1/J_{min}$, J = 120 Hz and $1/J_{max}$, J = 170 Hz; Long-range coupling delay d6, 83 ms ($^1\!/_{2Jlr}$, J = 6 Hz). The total experiment time was 6.46 h. Processing to 2k × 1k datapoints used matched Gaussian apodization (LB = −0.30, GB = 83/174 = 0.479 in F2; LB = −0.50, GB = 0.001 in F1) without linear prediction.

Figure S9C. Spectrum and parameters are as for Figure 3 on fully-labeled poplar stem EL; a slightly wider carbon-range (170-100 ppm) is plotted here (to accommodate other peaks below carbon *p***HB**$_7$ in the HMBC spectrum that are not relevant here). The spectrum was acquired with 8 scans per increment, a 3 s interscan delay, and had a total experiment time of 7.11 h.

PULSEPROGRAMS

A) CT-HSQC pulseprogram (Marco Tonelli’s version, minor editing by JR; based on Bruker’s hsqcctetgpsisp pulseprogram)

```
;hsqcctetgpsisp_jr.mt
;avance-version (15/02/27)
;HSQC
;2D H-1/X correlation via double inept transfer
;   using sensitivity improvement
;phase sensitive using Echo/Antiecho-TPPI gradient selection
;with decoupling during acquisition
;constant time version
;using trim pulses in inept transfer
;using shaped pulses for inversion on f2 - channel
;
;(G.W. Vuister & A. Bax, J. Magn. Reson. 98, 428-435 (1992))
;(A.G. Palmer III, J. Cavanagh, P.E. Wright & M. Rance, J. Magn.
;   Reson. 93, 151-170 (1991))
;
;$CLASS=HighRes
;$DIM=2D
;$TYPE=
;$SUBTYPE=
;$COMMENT=

prosol relations=<triple>

#include <Avance.incl>
#include <Grad.incl>
#include <Delay.incl>

“p2=p1*2”
“p4=p3*2”
“p22=p21*2”
“d4=1s/(cnst2*4)”
“d11=30m”
“d12=20u”
“d13=4u”

“d0=3u”
“d20=d23-p16-d16”
“in0=inf1/2”
“in20=in0”

“td1=tdmax(td1,d20*2,in20)”

“DELTA1=p16+d16-p1*0.78+de+8u”
“DELTA2=d4-larger(p2,p14)/2-d13-p16”
“DELTA3=d23-p2-d0-p16-d16”
“DELTA4=d24-p19-d13”
“DELTA12=d4-larger(p2,p14)/2-p16-d16”
“DELTA14=d24-p19-d16”

;;”d21=trunc(d20/in20)*2”
;d21: max number of 13C points that can be used
“d21=(d20/in20)*2”

“spoffs3=0”
; “spoffs5=bf2*(cnst21/1000000)-o2”
“spoffs13=0”

;p17: clean up gradient
;;”p17=3.5m”                    ; strong gradient if you need for water suppression
                                ; can make it shorter (1ms) if not needed for water suppression
```

```
"acqt0=0"
baseopt_echo


1 ze
  d11 pl12:f2
2 d1 do:f2
  50u UNBLKGRAD
3 (p1 ph1)
  d13
  p16:gp6
  DELTA2 pl0:f2
;  4u
  (center (p2 ph1) (p8:sp13 ph6):f2 )
;  4u
  DELTA12 pl2:f2
  p16:gp6
  d16
  (p1 ph2)
;;;
  d13
  p17:gp5
  d16
;;;
  (p3 ph3):f2
;;;;;;;;;;;;;;;;;;;;;;;;;
; C13 EVOLUTION BEGINS
;;;;;;;;;;;;;;;;;;;;;;;;;
  d0
  (p2 ph7)
  DELTA3
  p16:gp11
  d16
;;;
  (p4 ph8):f2
;;;
  d20
  p16:gp1
  d16
;;;;;;;;;;;;;;;;;;;;;;;;;
; C13 EVOLUTION ENDS
;;;;;;;;;;;;;;;;;;;;;;;;;
  (center (p1 ph1) (p3 ph4):f2 )
  d13
  p19:gp3
  DELTA4
  (center (p2 ph1) (p4 ph1):f2 )
  DELTA14
  p19:gp3
  d16
  (center (p1 ph2) (p3 ph5):f2 )
  d13
  p16:gp4
  DELTA2 pl0:f2
  (center (p2 ph1) (p8:sp13 ph1):f2 )
  DELTA12
  p16:gp4
  d16
  (p1 ph1)
  DELTA1
  (p2 ph1)
  4u
  p16:gp2*EA
  d16 pl12:f2
  4u BLKGRAD
  go=2 ph31 cpd2:f2
  d1 do:f2 mc #0 to 2
```

```
  F1EA(calgrad(EA) & calph(ph5, +180), caldel(d0, +in0) & caldel(d20, -in20) & calph(ph3, +180) & calph(ph6, +180) & calph(ph31, +180))

  if "d21>0" \n{\n 2u \n}         ;;; max number of 13C increments

exit

ph1 =0
ph2 =1
ph3 =0 2
ph4 =0 0 2 2
ph5 =1 1 3 3
ph6 =0
ph7 =0 0 2 2
ph8 =0 0 0 0 1 1 1 1 2 2 2 2 3 3 3 3
ph31=0 2 2 0 2 0 0 2

;pl0 : 0W
;pl1 : f1 channel - power level for pulse (default)
;pl2 : f2 channel - power level for pulse (default)
;pl3 : f3 channel - power level for pulse (default)
;pl12: f2 channel - power level for CPD/BB decoupling
;sp3 : f2 channel - shaped pulse 180 degree (on resonance)
;sp5 : f2 channel - shaped pulse 180 degree (off resonance)
;sp13: f2 channel - shaped pulse 180 degree (adiabatic)
;p1 : f1 channel -  90 degree high power pulse
;p2 : f1 channel - 180 degree high power pulse
;p3 : f2 channel -  90 degree high power pulse
;p4 : f2 channel - 180 degree high power pulse
;p8 : f2 channel - 180 degree shaped pulse for inversion (adiabatic)
;p14: f2 channel - 180 degree shaped pulse
;p16: homospoil/gradient pulse
;p22: f3 channel - 180 degree high power pulse
;p28: f1 channel - trim pulse                    [1 msec]
;d0 : incremented delay (2D)                     [3 usec]
;d1 : relaxation delay; 1-5 * T1
;d4 : 1/(4J)XH
;d11: delay for disk I/O                         [30 msec]
;d12: delay for power switching                  [20 usec]
;d16: delay for homospoil/gradient recovery
;d20 : = d23
;d23: d23 = T : 13.3 or 26.6 msec
;     2T (constant time period) = n/J(CC)
;d24: 1/(8J)XH for all multiplicities
;     1/(4J)XH for XH
;cnst2: = J(XH)
;cnst21: CO chemical shift (offset, in ppm)
;inf1: 1/SW(X) = 2 * DW(X)
;in0: 1/(2 * SW(X)) = DW(X)
;in20: = in0
;nd0: 2
;ns: 8 * n
;ds: 32
;td1: number of experiments
;FnMODE: echo-antiecho
;cpd2: decoupling according to sequence defined by cpdprg2
;pcpd2: f2 channel - 90 degree pulse for decoupling sequence

;for z-only gradients:
;gpz1:  -87%          : encoding gradient (NOTE: gpz1-gpz11 must be -80%)
;gpz11: -7%           : encoding gradient (NOTE: gpz1-gpz11 must be -80%)
;gpz2:  20.1% for C-13, 8.1% for N-15  : decoding gradient
;gpz5:  67% - use strong gradient if you need for water suppression
;          can make weaker if not needed for water suppression
;                       (e.g. 29% - this is just a clean up gradient, so its
;                       strength and duration is not bound to any other gradient)
```

;use gradient files:
;gpnam1: SMSQ10.100
;gpnam2: SMSQ10.100

;$Id: hsqcctetgpsisp,v 1.9.2.1 2015/03/03 11:21:23 ber Exp $

B) CT-HSQC-TOCSY pulseprogram (Marco Tonelli’s version)

```
;hsqcdictetgpsisp.mt2
;avance-version (15/02/27)
;HSQC
;2D H-1/X correlation via double inept transfer
;   using sensitivity improvement
;phase sensitive using Echo/Antiecho-TPPI gradient selection
;with decoupling during acquisition
;constant time version
;using trim pulses in inept transfer
;using shaped pulses for inversion on f2 - channel
;
;(G.W. Vuister & A. Bax, J. Magn. Reson. 98, 428-435 (1992))
;(A.G. Palmer III, J. Cavanagh, P.E. Wright & M. Rance, J. Magn.
;   Reson. 93, 151-170 (1991))
;
;$CLASS=HighRes
;$DIM=2D
;$TYPE=
;$SUBTYPE=
;$COMMENT=

prosol relations=<triple>

#include <Avance.incl>
#include <Grad.incl>
#include <Delay.incl>

“p2=p1*2”
“p4=p3*2”
“p22=p21*2”
“d4=1s/(cnst2*4)”
“d11=30m”
“d12=20u”
“d13=4u”

/************************************/
/* calculate DISPI number of cycles */
;d9 : TOCSY mixing time (sec)
;p6 : f1 channel -  90 degree pulse for TOCSY mixing
;pl10 : f1 channel - power level for TOCSY mixing
“FACTOR1=(d9/(p6*115.112))/2”
“l1=FACTOR1*2”
/************************************/

“d0=3u”
“in0=inf1/2”
“in20=in0”

“DELTA1=p16+d16-p1*0.78+de+8u”
“DELTA2=d4-larger(p2,p14)/2-d13-p16”
“DELTA12=d4-larger(p2,p14)/2-p16-d16”
“DELTA4=d24-p19-d13”
“DELTA14=d24-p19-d16”

#   ifdef decouple_CO
“DELTA3=d23-d0-larger(p14,p22)-p16-d16”
“d20=d23-p16-d16-p14-4u-d12”
#   else
“DELTA3=d23-d0-p22-p16-d16”
“d20=d23-p16-d16”
#   endif /*decouple_CO*/

“td1=tdmax(td1,d20*2,in20)”
```

```
"spoffs3=0"
"spoffs5=bf2*(cnst21/1000000)-o2"
"spoffs13=0"

"p17=3.5m"                          ; strong gradient for water suppression

"acqt0=0"
baseopt_echo


1 ze
  d11 pl12:f2
  d11 pl16:f3

2 d1 do:f2 do:f3
  d11 pl3:f3
  50u UNBLKGRAD

3 (p1 ph1)
  d13
  p16:gp6
  DELTA2 pl0:f2
  (center (p2 ph1) (p8:sp13 ph6):f2 )
  DELTA12 pl2:f2
  p16:gp6
  d16
  (p1 ph2)
;;;
  d13
  p17:gp5
  d16
;;;
  (p3 ph3):f2
;;;;;;;;;;;;;;;;;;;;;;;;
; C13 EVOLUTION BEGINS
;;;;;;;;;;;;;;;;;;;;;;;;
  d0

#   ifdef decouple_CO
  (center (p2 ph7) (p14:sp5 ph1):f2 (p22 ph1):f3 )
  DELTA3
  p16:gp11
  d16 pl2:f2
#   else
  (center (p2 ph7) (p22 ph1):f3 )
  DELTA3
  p16:gp11
  d16
#   endif /*decouple_CO*/

;;;
  (p4 ph8):f2
;;;
  d20
  p16:gp1

#   ifdef decouple_CO
  d16 pl0:f2
  (p14:sp5 ph1):f2
  4u
  d12 pl2:f2
#   else
  d16
#   endif /*decouple_CO*/

;;;;;;;;;;;;;;;;;;;;;;;;
; C13 EVOLUTION ENDS
```

```
;;;;;;;;;;;;;;;;;;;;;;;;
 (center (p1 ph1) (p3 ph4):f2 )
;;;
 d13
 p19:gp3
 DELTA4
 (center (p2 ph1) (p4 ph1):f2 )
 DELTA14
 p19:gp3
 d16
;;;
 (center (p1 ph2) (p3 ph5):f2 )
;;;
 d13
 p16:gp4
 DELTA2 pl0:f2
 (center (p2 ph1) (p8:sp13 ph1):f2 )
 DELTA12
 p16:gp4
 d16 pl10:f1
;;;
; TOCSY STARTS
;;;
                                                  ;begin DIPSI2
4 p6*3.556 ph22
 p6*4.556 ph24
 p6*3.222 ph22
 p6*3.167 ph24
 p6*0.333 ph22
 p6*2.722 ph24
 p6*4.167 ph22
 p6*2.944 ph24
 p6*4.111 ph22

 p6*3.556 ph24
 p6*4.556 ph22
 p6*3.222 ph24
 p6*3.167 ph22
 p6*0.333 ph24
 p6*2.722 ph22
 p6*4.167 ph24
 p6*2.944 ph22
 p6*4.111 ph24

 p6*3.556 ph24
 p6*4.556 ph22
 p6*3.222 ph24
 p6*3.167 ph22
 p6*0.333 ph24
 p6*2.722 ph22
 p6*4.167 ph24
 p6*2.944 ph22
 p6*4.111 ph24

 p6*3.556 ph22
 p6*4.556 ph24
 p6*3.222 ph22
 p6*3.167 ph24
 p6*0.333 ph22
 p6*2.722 ph24
 p6*4.167 ph22
 p6*2.944 ph24
 p6*4.111 ph22
 lo to 4 times l1
                                                  ;end DIPSI2
 4u pl1:f1
;;;
```

```
  (p1 ph1)
;;;
  DELTA1
  (p2 ph1)
  4u
  p16:gp2*EA
  d16 pl12:f2 pl16:f3
  4u BLKGRAD
;;;
#   ifdef decouple_N15
  go=2 ph31 cpd2:f2 cpd3:f3
#   else
  go=2 ph31 cpd2:f2
#   endif /*decouple_N15*/
  d1 do:f2 do:f3 mc #0 to 2
     F1EA(calgrad(EA) & calph(ph5, +180), caldel(d0, +in0) & caldel(d20, -in20) & calph(ph3, +180) & calph(ph6, +180) & calph(ph31, +180))
exit


ph1 =0
ph2 =1
ph3 =0 2
ph4 =0 0 2 2
ph5 =1 1 3 3
ph6 =0
ph7 =0 0 2 2
ph8 =0 0 0 0 1 1 1 1 2 2 2 2 3 3 3 3
ph31=0 2 2 0 2 0 0 2

/* TOCSY phases */
ph22=3
ph24=1
/****************/


;pl0 : 0W
;pl1 : f1 channel - power level for pulse (default)
;pl2 : f2 channel - power level for pulse (default)
;pl3 : f3 channel - power level for pulse (default)
;pl12: f2 channel - power level for CPD/BB decoupling
;sp3 : f2 channel - shaped pulse 180 degree (on resonance)
;sp5 : f2 channel - shaped pulse 180 degree (off resonance)
;sp13: f2 channel - shaped pulse 180 degree (adiabatic)
;p1 : f1 channel -  90 degree high power pulse
;p2 : f1 channel - 180 degree high power pulse
;p3 : f2 channel -  90 degree high power pulse
;p4 : f2 channel - 180 degree high power pulse
;p8 : f2 channel - 180 degree shaped pulse for inversion (adiabatic)
;p14: f2 channel - 180 degree shaped pulse
;p16: homospoil/gradient pulse
;p22: f3 channel - 180 degree high power pulse
;p28: f1 channel - trim pulse                    [1 msec]
;d0 : incremented delay (2D)                     [3 usec]
;d1 : relaxation delay; 1-5 * T1
;d4 : 1/(4J)XH
;d11: delay for disk I/O                         [30 msec]
;d12: delay for power switching                  [20 usec]
;d16: delay for homospoil/gradient recovery
;d20 : = d23
;d23: d23 = T : 13.3 or 26.6 msec
;     2T (constant time period) = n/J(CC)
;d24: 1/(8J)XH for all multiplicities
;     1/(4J)XH for XH
;cnst2: = J(XH)
;cnst21: CO chemical shift (offset, in ppm)
;inf1: 1/SW(X) = 2 * DW(X)
```

```
;in0: 1/(2 * SW(X)) = DW(X)
;in20: = in0
;nd0: 2
;ns: 4 * n
;ds: 32
;td1: number of experiments
;FnMODE: echo-antiecho
;cpd2: decoupling according to sequence defined by cpdprg2
;pcpd2: f2 channel - 90 degree pulse for decoupling sequence


;use gradient ratio:    gp 1 : gp 2
;                       80 : 20.1    for C-13
;                       80 :  8.1    for N-15

;for z-only gradients:
;gpz1: 80%
;gpz2: 20.1% for C-13, 8.1% for N-15
;gpz5: 67% - use strong gradient for water suppression

;use gradient files:
;gpnam1: SMSQ10.100
;gpnam2: SMSQ10.100


;$Id: hsqcctetgpsisp,v 1.9.2.1 2015/03/03 11:21:23 ber Exp $
```

C) C–C-FLOPSY pulseprogram (Guy Lippens' version, based on the 2003 Eletsky, Moreira, Kovacs, and Pervushin[55])

```
;c_ccflopsy16
;avance-version
;CC-TOCSY
;2D sequence with
;   homonuclear Hartman-Hahn transfer using FLOPSY16
;      sequence for mixing
;phase sensitive (t1)
;
;M. Kadkhodaie, O. Rivas, M. Tan, A. Mohebbi, A.J. Shaka,
;    J. Magn. Res. 91, 437-443 (1991)
;A. Eletsky, O.Moreira, H. Kovacs & K. Pervushin,
;   J. Biomol. NMR 26, 167-179 (2003)
;Modified GL 28/09/23 to remove 13CO refocusing, 15N decoupling
;
;$CLASS=HighRes
;$DIM=2D
;$TYPE=
;$SUBTYPE=
;$COMMENT=

prosol relations=<triple_c>

#include <Avance.incl>
#include <Delay.incl>
#include <Grad.incl>

"p2=p1*2"
"d11=30m"
"d12=20u"
"d0=3u"
"in0=inf1/2"

"FACTOR1=(d9/(p6*188.448))"
"l1=FACTOR1"

1 ze
  d11 pl12:f2
2 d11 do:f2
3 d1 pl1:f1
  50u UNBLKGRAD
  d12 cpd2:f2

  (p1 ph3)
  d0
  d0
  (p1 ph4)

  4u do:f2
  p16:gp1
  d16 pl10:f1

;begin FLOPSY16
4 p6*0.511 ph11
  p6*1.067 ph12
  p6*1.822 ph13
  p6*1.767 ph14
  p6*1.444 ph15
  p6*1.767 ph14
  p6*1.822 ph13
  p6*1.067 ph12
  p6*0.511 ph11

  p6*0.511 ph21
  p6*1.067 ph22
```

p6*1.822 ph23
p6*1.767 ph24
p6*1.444 ph25
p6*1.767 ph24
p6*1.822 ph23
p6*1.067 ph22
p6*0.511 ph21

p6*0.511 ph21
p6*1.067 ph22
p6*1.822 ph23
p6*1.767 ph24
p6*1.444 ph25
p6*1.767 ph24
p6*1.822 ph23
p6*1.067 ph22
p6*0.511 ph21

p6*0.511 ph11
p6*1.067 ph12
p6*1.822 ph13
p6*1.767 ph14
p6*1.444 ph15
p6*1.767 ph14
p6*1.822 ph13
p6*1.067 ph12
p6*0.511 ph11

p6*0.511 ph11
p6*1.067 ph12
p6*1.822 ph13
p6*1.767 ph14
p6*1.444 ph15
p6*1.767 ph14
p6*1.822 ph13
p6*1.067 ph12
p6*0.511 ph11

p6*0.511 ph11
p6*1.067 ph12
p6*1.822 ph13
p6*1.767 ph14
p6*1.444 ph15
p6*1.767 ph14
p6*1.822 ph13
p6*1.067 ph12
p6*0.511 ph11

p6*0.511 ph21
p6*1.067 ph22
p6*1.822 ph23
p6*1.767 ph24
p6*1.444 ph25
p6*1.767 ph24
p6*1.822 ph23
p6*1.067 ph22
p6*0.511 ph21

p6*0.511 ph21
p6*1.067 ph22
p6*1.822 ph23
p6*1.767 ph24
p6*1.444 ph25
p6*1.767 ph24
p6*1.822 ph23
p6*1.067 ph22
p6*0.511 ph21

p6*0.511 ph21
p6*1.067 ph22
p6*1.822 ph23
p6*1.767 ph24
p6*1.444 ph25
p6*1.767 ph24
p6*1.822 ph23
p6*1.067 ph22
p6*0.511 ph21

p6*0.511 ph11
p6*1.067 ph12
p6*1.822 ph13
p6*1.767 ph14
p6*1.444 ph15
p6*1.767 ph14
p6*1.822 ph13
p6*1.067 ph12
p6*0.511 ph11

p6*0.511 ph11
p6*1.067 ph12
p6*1.822 ph13
p6*1.767 ph14
p6*1.444 ph15
p6*1.767 ph14
p6*1.822 ph13
p6*1.067 ph12
p6*0.511 ph11

p6*0.511 ph21
p6*1.067 ph22
p6*1.822 ph23
p6*1.767 ph24
p6*1.444 ph25
p6*1.767 ph24
p6*1.822 ph23
p6*1.067 ph22
p6*0.511 ph21

p6*0.511 ph21
p6*1.067 ph22
p6*1.822 ph23
p6*1.767 ph24
p6*1.444 ph25
p6*1.767 ph24
p6*1.822 ph23
p6*1.067 ph22
p6*0.511 ph21

p6*0.511 ph21
p6*1.067 ph22
p6*1.822 ph23
p6*1.767 ph24
p6*1.444 ph25
p6*1.767 ph24
p6*1.822 ph23
p6*1.067 ph22
p6*0.511 ph21

p6*0.511 ph11
p6*1.067 ph12
p6*1.822 ph13
p6*1.767 ph14
p6*1.444 ph15
p6*1.767 ph14

```
  p6*1.822 ph13
  p6*1.067 ph12
  p6*0.511 ph11

  p6*0.511 ph11
  p6*1.067 ph12
  p6*1.822 ph13
  p6*1.767 ph14
  p6*1.444 ph15
  p6*1.767 ph14
  p6*1.822 ph13
  p6*1.067 ph12
  p6*0.511 ph11
  lo to 4 times l1
;end FLOPSY16
  p16:gp2
  d16 pl1:f1
  4u BLKGRAD
  (p1 ph5)

  go=2 ph31 cpd2:f2
  d11 do:f2  mc #0 to 2
     F1PH(calph(ph3, +90), caldel(d0, +in0))
exit

ph1=0
ph3=0 2
ph4=0 0 0 0 2 2 2 2
ph5=0 0 2 2

ph11=(720) 0
ph12=(720) 90
ph13=(720) 135
ph14=(720) 630
ph15=(720) 45

ph21=(720) 360
ph22=(720) 450
ph23=(720) 495
ph24=(720) 270
ph25=(720) 405

ph31=0 2 2 0 2 0 0 2

;pl1 : f1 channel - power level for pulse (default)
;pl10: f1 channel - power level for TOCSY-spinlock
;pl12: f2 channel - power level for CPD/BB decoupling
;pl16: f3 channel - power level for CPD/BB decoupling
;sp26: f1 channel - shaped pulse 180 degree  (C=O off resonance)
;p1 : f1 channel -  90 degree high power pulse
;p2 : f1 channel - 180 degree high power pulse
;p6 : f1 channel -  90 degree low power pulse        [25 usec]
;p12: f1 channel - 180 degree shaped pulse
;p16: homospoil/gradient pulse              [1 msec]
;d0 : incremented delay (F1 in 2D)         [3 usec]
;d1 : relaxation delay; 1-5 * T1
;d9 : FLOPSY mixing time and the 13C B1 field employed for the pulse train     [20 msec]
;d11: delay for disk I/O           [30 msec]
;d12: delay for power switching             [20 usec]
;d16: delay for homospoil/gradient recovery
;cnst21: CO chemical shift (offset, in ppm) – removed, not relevant here!
;o1p: Caliphatic chemical shift (cnst23) – removed, not relevant here!
;l1: loop for FLOPSY16 cycle: ((p6*188.448) * l1) = mixing time
;inf1: 1/SW(Cali) = 2 * DW(Cali)
;in0: 1/(2 * SW(Cali)) =  DW(Cali)
;nd0: 2
;ns: 8 * n
```

```
;ds: >= 32
;td1: number of experiments in F1
;FnMODE: States-TPPI (or TPPI) in F1
;cpd2: decoupling according to sequence defined by cpdprg2
;cpd3: decoupling according to sequence defined by cpdprg3
;pcpd2: f2 channel - 90 degree pulse for decoupling sequence
;pcpd3: f3 channel - 90 degree pulse for decoupling sequence

;use gradient ratio:    gp 1 : gp 2
;                         80 :   70

;for z-only gradients:
;gpz1: 80%
;gpz2: 70%

;use gradient files:
;gpnam1: SMSQ10.100
;gpnam2: SMSQ10.100

;$Id:$
```
